# How I See Me—A Meta-Analysis Investigating the Association Between Identities and Pro-environmental Behaviour

**DOI:** 10.3389/fpsyg.2021.582421

**Published:** 2021-03-16

**Authors:** Alina Mia Udall, Judith I.M. de Groot, Simon B. De Jong, Avi Shankar

**Affiliations:** ^1^Warwick Business School, University of Warwick, Coventry, United Kingdom; ^2^School of Engineering, Newcastle University, Newcastle upon Tyne, United Kingdom; ^3^Department of Marketing, Faculty of Economics and Business, University of Groningen, Grongingen, Netherlands; ^4^Department of Organization, Strategy, and Entrepreneurship, School of Business and Economics, Maastricht University, Maastricht, Netherlands; ^5^Department of Marketing, Centre for Business, Organisations and Society, University of Bath, Bath, United Kingdom

**Keywords:** identities, pro-environmental behaviour, group identity, methods—estimation, individual identification, climate change, sustainability, meta-analysis

## Abstract

Prolific research suggests identity associates with pro-environmental behaviours (PEBs) that are individual and/or group focused. Individual PEB is personally driven, self-reliant, and are conducted on one's own (e.g., home recycling). Group focused PEB is other people-reliant and completed as part of a group (e.g., attending meetings of an environmental organisation). A wide range of identities have been related to PEBs. For example, a recent systematic qualitative review revealed 99 different types of identities studied in a PEB context. Most studies were correlational, few had an experimental design. However, the relationships between all these identities and PEBs have so far not been tested quantitatively with meta-analytical techniques. As such, a clear overview of this field is currently lacking. Due to the diverse nature of the field, *a priori* hypotheses were not possible and relatively broad definitions of identity had to be used to encompass all types of identities and the diverse meanings of identity that have been included in PEB research. What prior theory did allow for was to assess the distinction between two main types of identity, namely how people label, describe, and recognise oneself individually (individual identity), or as part of a group (group identity). Our overall goal was thus to assess the current state of knowledge on identities and PEBs. In 104 studies using a meta-regression following the preferred reporting items for systematic reviews and meta-analyses guidelines, our random-effects meta-analysis showed that the overall concept of identity associated with PEB with a medium Pearson's *r* (Aim 1). Furthermore, we found that individual identities associated more strongly with PEBs than group identities (Aim 2). The associations between individual and group identities were stronger when the identity and PEB were from the same category (e.g., when both were group-focused; Aim 3). Methodologically, the findings revealed that group identities and group PEBs were most strongly associated for self-reported rather than observed PEBs (Aim 4). Overall identity associated most strongly with group PEBs in the field rather than in the lab (Aim 5) and in student- rather than non-student samples (Aim 6). We discuss the theoretical and practical implications.

## Introduction

Humans generate many environmental problems via behaviours which are not sustainable in the longer term (Allen et al., 2018). Consequently, there is an urgent need for researchers to understand how to encourage people to behave in a pro-environmental way. Pro-environmental behaviour (PEB) is defined as actions that minimise the negative impact on (such as preserving and preventing damage to), and/or promoting improvements to, the natural and the built world (Kollmuss and Agyeman, 2002). People can carry out individual PEB which is largely personally driven. It is primarily an action that is self-reliant and driven by the self. In particular, it is a behaviour that is conducted on one's own, for example home recycling. Alternatively, people can carry out group PEB which is behaviour that is carried out as part of a group, for example, attending meetings of an environmental organisation.

There has been a recent surge of research on understanding how to encourage PEBs (Chernev and Blair, 2015; Gershoff and Frels, 2015; Brick et al., 2017; Walton and Jones, 2017; Brick and Lai, 2018). Specifically, a meta-analysis showed that research in this field was largely based on four dominant psychological theories (Klöckner, 2013, see also Van Den Broek and Walker, 2019), namely the theory of planned behaviour (Ajzen, 1991), the norm-activation theory (Schwartz, 1992), the value-belief-norm theory (Stern, 2000), and habits (Verplanken and Aarts, 1999; Verplanken and Ryan, 2018). However, combining these four theories accounted for only 36% of variance explained in a variety of PEBs (Klöckner, 2013). We argue that the explanatory power of these models may be improved by including identity.

Generally, identity refers to how people see themselves (Pronin, 2008). While research has emerged to incorporate identity into the most popular theories used to explain PEBs, such as the theory of planned behaviour (Pierro et al., 2003; Fielding et al., 2008a; Fielding and Hornsey, 2016; see meta-analysis by Rise et al., 2010), identities seem to be important independent from these theories as well (Murtagh et al., 2012). Indeed, identity in PEB research has blossomed over the last three decades (Dagher and Itani, 2014; Graham-Rowe et al., 2015; Walton and Jones, 2017; Hamerman et al., 2018; Brieger, 2019). A recent systematic literature review showed that 99 different identities have been studied in a PEB context (Udall et al., 2020), such as pro-environmental identity, moral identity, and social identity. Some of these identities have been studied in experimental designs, while others have taken correlational designs. However, there is no meta-analysis to show the extent to which this multitude of identities studied in relation to PEB are actually associated with PEB. Therefore, the main aim of the present study is to consolidate the PEB research by empirically testing the relationships of these identities to PEB. More specifically, we will aim to provide a structure within the diffused research on identities and PEBs by ordering them in two theoretically meaningful ways: Making a distinction between individual and group identities, and, individual and group PEBs.

Identities can be categorised as either an individual or group identity which originates from the self-categorisation theory (Turner et al., 1987), a key psychological identity theory. An individual identity is defined as how people label, describe, and recognise oneself individually or personally (e.g., environmental identity). A group-identity is defined as how people label, describe, and recognise themselves as part of a group (e.g., environmental group identity). The literature on identity and PEB has not only identified 99 different types of identity but has also examined these identities in a variety of PEBs which can also be meaningfully distinguished between individual or group types (Udall et al., 2020). The distinction between individual and group PEBs as well as identities allow us to not only explore the importance of identity in relation to PEB in general, but to understand the extent to which these different identities per category associate with these PEBs per category. Hence, unifying the diverse conceptualisations of identity and PEB in the field can contribute to further consolidate the diverse field. Furthermore, as the field seems to have taken quite different methodological approaches to investigate the identity-PEB relationship, we also check how three key methodological choices may affect this association. More specifically, the present study explores how the measurement of PEBs (self-reported or observed), using different research designs (lab or field), and using student or non-student samples (Kormos and Gifford, 2014) moderate the identity and PEB associations.

Based on the prolific study of identity in PEB research, and the diffuse nature of these studies, the present study proposes six broad aims (rather than *a priori* hypotheses) to assess the literature quantitatively to consolidate this topic (Thomas et al., 2019). This approach enables us to provide a comprehensive quantitative summary of the evidence, which is currently lacking, yet which is important for the advancement of a field (which has been the case in, for example, predicting crime; Walters, 2020). Also, we can explore consistency, and therefore generalisability, of findings across different types of research on this topic which is yet to be completed (Thomas et al., 2019; Walters, 2020). Our meta-analysis will therefore be an exploration following the preferred reporting items for systematic reviews and meta-analyses (PRISMA) guidelines (Moher et al., 2009). From this meta-analysis, we deduce future directions and practical implications about how to assess identities alongside other psychological variables in PEB research. We will introduce the relevance of the six aims in relation to state-of-the-art of the literature in the field of identity and PEB.

### Aim 1: Associations Between Overall Identity and PEB

Identity theory (Stryker and Burke, 2000) and social identity theory (Tajfel and Turner, 1979) are dominant psychological theories that explicitly deal with how identity relates to behaviour generally and to PEB specifically (Udall et al., 2020). Identity theory suggests that when an individual identity is in the foreground or “salient,” this identity will more likely associate with behaviour when the meaning of the behaviour corresponds to the meaning of the identity (Stryker and Burke, 2000)—and that either individual or group behaviours can be associated with it. Social identity theory assumes that if a person identifies with a specific group (salient group identity), they internalise the norms of the group and are therefore more likely to act in accordance with those norms (Tajfel and Turner, 1979). On the contrary, Udall et al.'s (2020) systematic literature review in identities and PEBs suggests that when an identity (individual or group) is in the foreground, the identity will associate with any PEB (either individual and/or group) as long as the meaning of the behaviour corresponds to the meaning of the identity. For example, people with a strong pro-environmental self-identity or pro-environmental group identity will carry out private nature conservation (individual PEB) and environmental activism (group PEB) because the meaning of the behaviours are PEB focused and correspond to the meaning of the identity's (also PEB focused). Therefore, the latter reasoning assumes that regardless of the type of identity and PEB (individual/group), they will associate with each other. However, it has never been explored.

Furthermore, the identity theories above imply that identity can associate with PEB in two ways. Firstly, identity associates with PEB because they incite schemas. Schemas are internally stored information about situations and expected behaviours, which are linked to that identity (Markus, 1977; Stryker and Burke, 2000). Secondly, identities incite norms (internally stored information about what is usual, typical, or standard in the given situation) toward the PEB in question (Cialdini et al., 1990). Understanding the extent to which schemas or norms explain the relationship between identity and PEB, we first need to gain consensus as to whether identity relates to PEB. There are currently many identity types measured in PEB research at the moment and related to many different types of PEBs, making it unclear which overall conclusions can be drawn. A meta-analysis enables us to reveal the inconsistencies within the literature about whether the prolific amount of identities overall associate (if at all) with PEB. Our meta-analysis will therefore not establish the causal mechanism, but it simply explores whether we can uncover a meaningful relationship to begin with. Therefore, Aim 1 focuses on how the overall concept of identity relates to PEBs in general.

### Aim 2: Associations Between Individual and Group Identities and Overall PEB

Identity can be either focused on the individual or group. However, the studies in PEB research usually have not made a distinction between individual and group identities explicitly (Udall et al., 2020). In line with this, they have largely ignored the theoretical assumptions that this distinction implies (Murtagh et al., 2012). For example, many different identity terms have been used to refer to somewhat the same individual identity (Whitmarsh and O'Neill, 2010; Bhattacharjee et al., 2014), such as an “ecological self-identity” (Castro et al., 2009), and “environmental identity” (Brügger et al., 2011; Tam, 2013). Furthermore, the same diverse terms have been used for group identities, for example, social identity (Costa-Pinto et al., 2014, 2016), social identity importance: “member of the local community” (Murtagh et al., 2012), and “environmental movement identity” (Dunlap and McCright, 2008). Such confusions in conceptualisation and operationalisation makes the literature in this field disparate. Structuring the multitude of identities as individual or group identities will help to understand the findings of each study in relation to identity theory. To provide structure, it is therefore useful to categorise the multitude of studied identity types.

Furthermore, research shows that the strength of the associations between individual and group identities on PEBs vary. For example, individual identities associate with PEBs, like an environmental identity (Hinds and Sparks, 2008), car-authority identity (Schuitema et al., 2013), and ecological self-identity (Castro et al., 2009). However, some group identities do not associate with PEBs like a social identity (Costa-Pinto et al., 2014, 2016), consumer's identification with a socially responsible insurance company (Pérez, 2009), environmental movement identity (Dunlap and McCright, 2008), and rural group identification (Fielding et al., 2008b). Comparing the extent to which individual and groups identities associate with PEB has never been explored. Our meta-analysis will therefore distinguish the multitude of identities studied in the field of PEB into individual and group identities. This distinction will help to understand the findings of each study in relation to the assumptions that have been made in popular identity theories. Therefore, Aim 2 focuses on how individual and group identities relates to PEBs overall.

### Aim 3: Associations Between Overall Identity and Individual and Group PEBs

Like identities, PEBs can also be categorised as either individual or group PEB, although studies usually have not made such distinction (Udall et al., 2020). The lack of categorising PEBs in identity research has resulted in confusion about the conceptualisations and measurement of PEBs. For example, many different individual PEBs have been used to refer to somewhat the same PEB (Whitmarsh and O'Neill, 2010; Matsuba et al., 2012), such as an “ecological behaviour” (Whitmarsh and O'Neill, 2010; Brügger et al., 2011) and “environmental behaviour” (Matsuba et al., 2012). While, the same diverse terms have been used for group PEBs, for example, “public environmental behaviour” (Matsuba et al., 2012) and “member of an environmental organisation” (Dunlap and McCright, 2008). Structuring the multitude of PEBs as individual or group PEBs will help to understand the findings of each study in relation to theories of identity. For example, identity theory (Stryker and Burke, 2000) suggests that when the shared meanings of the identity and PEB are most similar, the associations are likely to be strongest. People with a strong “self-identity toward private nature conservation” (an individual identity) will more likely carry out individual PEB, such as engaging in private nature conservation (Lokhorst et al., 2014). Conversely, people with a strong group identity such as “a member of an environmental group” will more likely carry out a group PEB such as engaging in environmental activism (Fielding et al., 2008a). Therefore, it is useful to categorise the multitude of studied PEBs in a similar way as identities. Our meta-analysis will distinguish between individual and group PEBs which enables us to explore whether there is a systematic pattern of results between the aforementioned established individual and group identities. This distinction will help to further explore the extent to which a similar two-way distinction in PEBs as used in identities might be helpful to provide order in the identity-PEB literature (rather than testing specific a priori hypotheses in relation to the alignment assumption as coined by identity theory). Therefore, Aim 3 focuses on how individual and group identities relate to individual and group PEBs.

### Aim 4-6: Measures, Research Setting, and Samples as Moderators

Research in identity and PEB has taken quite different methodological approaches to investigate the identity-PEB relationship. In particular, the way in which PEB has been measured (Aim 4), the type of research design (Aim 5), and the sample (Aim 6) are three key moderators that may affect the identity-PEB associations.

Identity-PEB research typically relies on two types of PEB measures, namely self-reported intention/behaviour (Williams et al., 2004, 2006; Fitzsimons et al., 2007), and objective/observed behaviour (Chandon et al., 2004; Williams et al., 2004; Conner et al., 2011). Past research suggests that these types of measures may moderate empirical relations (Nigbur et al., 2010; Bamberg et al., 2015; Reese and Kohlmann, 2015). For example, identity-PEB associations were significant for self-reported PEB and not significant for observed PEBs (Reese and Kohlmann, 2015), or, they were at least stronger for self-reported PEBs (Spence et al., 2009; Nigbur et al., 2010). However, other studies have shown that the empirical associations are sometimes weaker for self-reported rather than observed measures (Sprott et al., 1999).

Identity-PEB associations may be moderated by measurement type because self-reported measures seem to be more susceptible to flaws in our ability to reliably reflect on our behaviour. For example, participants may exaggerate (Kormos and Gifford, 2014) or over-estimate their PEB (Geller, 1981; Warriner et al., 1984; Barr, 2007) due to a self-serving bias (Tarrant and Cordell, 1997). These biases are likely to occur as self-reports rely on reflecting on our memory of past behaviour, which can be easily re-written (Loftus and Palmer, 1974). To establish the role of PEB measures, Aim 4 focuses on how PEB measurements (self-reported and observed) moderate identity-PEB associations. Exploring the impact of measures on the identity-PEB relationship will help further understand the mixed empirical identity-PEB relationships, rather than understand which measure increases/decreases the identity-PEB associations.

Identity-PEB research is mostly conducted in either the laboratory (Crimston et al., 2016) or the field (Trump et al., 2015). Empirical associations may be strongest in a laboratory setting due to demand characteristics (Wood et al., 2015). People may feel pressure to act pro-environmentally in line with their identity because of the presence of the experimenter or the authority of the setting. However, the extent to which the research setting moderates the strength of this relationship is still unknown. To establish the role of research setting, Aim 5 focuses on how research settings (laboratory and field) moderate identity-PEB associations. Exploring the impact of settings on the identity-PEB relationships will help further understand the associations, rather than understand which setting increases/decreases the identity-PEB associations.

Finally, in PEB research usually two types of research samples are used, namely, student or non-student samples. Reliance on student samples has been criticised in psychological research as they do not reflect a generalised sample of the population at large and provide more positive results as students are more impressionable (Henrich et al., 2010). However, the extent to which the sample moderates the strength of this relationship is still missing. Therefore, Aim 6 focuses on how samples (students and non-students) moderate identity-PEB associations. Exploring the impact of samples on the identity-PEB relationships will help to further understand the identity-PEB findings, rather than understanding which setting increases/decreases the identity-PEB associations.

## Method

We used the PRISMA method (Moher et al., 2009), a widely used method, especially in the medical sciences (Drubbel et al., 2014; Holden et al., 2014). PRISMA offers a concise and replicable standard for conducting and reporting meta-analyses by advocating several reproducible steps (Higgins and Green, 2011), which we outline below.

### Protocol

A peer-reviewed protocol is necessary prior to the meta-analysis. This protocol was pre-registered on the Open Science Framework^1^. There were three changes between the pre-registered and the eventual methods and analysis. Firstly, in the pre-registration, we referred to “sustainable consumer behaviour” (SCB), rather than “PEB.” Both terms were used as search terms. Therefore, this only alters the narrative of the report, rather than the method and analysis. Secondly, after undertaking the initial review phase, we found further moderators that became pertinent to the study of identity in PEB, which were not included in the pre-registration. These include behavioural measure (observed and self-reported), study design (lab or field), and sample type (student or non-student). Additionally, we removed behaviour visibility as a moderator, because after collating the data, we found that there was insufficient information available to determine the visibility of many of the behaviours reported in the literature.

### Eligibility Criteria

Based on the main aims, in terms of populations, interventions, comparators, outcomes, and study designs (PICOS), we were interested in including all research that measured identity in relation to PEB, regardless of identity type (e.g., all individually focused identities: environmental identity, personal identity; all group focused identities: environmental group identity, social identity), outcome PEB type (i.e., self-reported intention, behaviour, observed PEB), study design type (i.e., correlational, quasi-experimental, experimental/interventional, within and/or between participants), comparator type (e.g., additional psychological variables assessed like habits—Verplanken and Ryan, 2018), or population type (e.g., student, non-student). These inclusion criteria were in line with our six aims. However, we used four additional criteria to answer these aims.

#### Criterion 1

The studies needed to examine identity and PEB empirically together. Studies needed to explicitly state that they examined identity and PEB or similar constructs that we interpreted as matching our definitions of identity and PEB, respectively. To clarify, eligible papers could capture any identity provided it was measured in relation to PEB. Furthermore, papers were eligible regardless of their definition of identity. When definitions were missing, and the term “identity” was not explicitly used, we checked if papers matched our definitions of identity before including them. For example, papers were included if they could be interpreted as matching our definition of the individual identity and/or group identity (Udall et al., 2020). In a similar way, in relation to PEB, papers were eligible if they explicitly stated that the behaviour was considered a PEB in line with our PEB definition (Kollmuss and Agyeman, 2002).

#### Criterion 2

Studies needed to use a design that allowed for the associations between identity and behaviour to be measured, compared, and obtained. Hence, quantitative studies were included and used in the analysis, such as correlational, experimental, or quasi-experimental data, but not qualitative studies.

#### Criterion 3

Studies needed to report statistics needed for the meta-analysis, such as means, standard deviations, odds ratios, correlation coefficients (*r*), Cohens *d*, partial eta squared, and other types of effect sizes enabling us to identify the strength of the identity-PEB associations. In line with previous research (Abrahamse and Steg, 2013), and due to the prevalence of correlational studies, we needed the effect size information that enabled us to convert any effect size to Pearson's *r* for consistency and comparability purposes (Pearson, 1895).

#### Criterion 4

We included primary studies that were published in peer-reviewed academic journals only. If data was missing from the published article, we contacted authors requesting this data, as well as any additional unpublished data they had (Rosenthal and DiMatteo, 2001; Field, 2005; Field and Wright, 2006; Abrahamse and Steg, 2013). We also contacted the authors to request additional information in relation to unreported statistics we needed. We attempted to gather the unpublished data pertaining to the published articles where possible. If these were not provided, we calculated the correlation coefficients, if sufficient data was available.

### Information Sources

We used the following electronic databases: (1) PsycArticles using PsycNet, (2) Web of Science, (3) EBSCOhost Business Source Complete, and (4) Scopus. Additional sources were Google Scholar alert and hand searching (Higgins and Green, 2011). Sources were searched from inception to 4th March 2016.

### Search Strategy

Using a modified checklist from the Cochrane Collaboration of Systematic Reviews (Higgins and Green, 2011), search terms and keywords were identified with search categories and filters (see Table 1). We chose search terms to maximise the identification of suitable articles and thus started with many search results. From that solid foundation we could then select the final, more focused, set of search results.

**Table 1:** Search terms for all databases with search categories and filters.

**PsycArticles using PsycNET APA**
Search terms: (“identity” AND “consumer behav*”) OR (“identity” AND “consum*”) OR (“identity” AND “green consumer behav*”) OR (“identity” AND “green consum*”) OR (“identity” AND “green behav*”) OR (“identity” AND “sustainable consumer behav*”) OR (“identity” AND “sustainable consum*”) OR (“identity” AND “sustainable behav*”) OR (“identity” AND “sustain*”) OR (“identity” AND “environmental consumer behav*”) OR (“identity” AND “environmental consum*”) OR (“identity” AND “environmental behav*”) OR (“identity” AND “environment*”) OR (“identity” AND “ecological consumer behav*”) OR (“identity” AND “ecological consum*”) OR (“identity” AND “ecological behav*”) OR (“identity” AND “eco*”) OR (“identity” AND “energy consumer behav*”) OR (“identity” AND “energy consum*”) OR (“identity” AND “energy behav*”) OR (“identity” AND “energy”) OR (“identity” AND “pro-environmental consumer behav*”) OR (“identity” AND “pro-environmental consum*”) OR (“identity” AND “pro-environmental behav*”) OR (“identity” AND “pro-environment*”) OR (“identity” AND “Proenvironmental consumer behav*”) OR (“identity” AND “Proenvironmental consum*”) OR (“identity” AND “Proenvironmental behav*”) OR (“identity” AND “proenvironment*”) OR (“identity” AND “environmentally friendly behav*”) OR (“identity” AND “environmentally friendly”) OR (“identity” AND “car use”) OR (“identity” AND “environmentally conscious consumer behav*”) OR (“identity” AND “environmentally conscious consum*”) OR (“identity” AND “environmentally conscious behav*”) OR (“identity” AND “environmentally conscious”) OR (“identity” AND “environmentally related consumer behav*”) OR (“identity” AND “environmentally related consum*”) OR (“identity” AND “environmentally related behav*”) OR (“identity” AND “environmentally related”) OR (“identity” AND “public transport consumer behav*”) OR (“identity” AND “public transport consum*”) OR (“identity” AND “public transport behav*”) OR (“identity” AND “public transport*”) OR (“identity” AND “waste recycling consumer behav*”) OR (“identity” AND “waste recycling consum*”) OR (“identity” AND “waste recycling behav*”) OR (“identity” AND “waste recycling”) OR (“identity” AND “recycling consumer behav*”) OR (“identity” AND “recycling consum*”) OR (“identity” AND “recycling behav*”) OR (“identity” AND “recycl*”) OR (“identity” AND “environmentally significant behav*”) OR (“identity” AND “ethical consumer behav*”) OR (“identity” AND “ethical consum*”) OR (“identity” AND “ethical behav*”) • Search category: In [Any Field]
**Web of science**
Same search terms as PsycArticles using PsycNET APA. No restrictions on search field. However, the following search terms were removed due to too many results: (“identity” AND “consum*”); (“identity” AND “sustain*”); (“identity” AND “environment*”); (“identity” AND “eco*”); (“identity” AND “energy”) • Search category: In [TOPIC]
**EBSCOhost business source complete** • Same search terms as Web of Science • Search category: In [Select a field (optional)]
**Scopus (Elsevier)** • The same search terms as Web of Science • Search category: Advanced Search box, which stated Search for…. • Also, the search was *limited to* four categories due to not being able to view more than 2000 results in this search engine. This enabled us to ensure that as many results that could be found were viewed. These categories were: (1) Document Type: Article, Short Survey, and Article in Press; (2) Keyword: Article, Priority Journal, Humans, Human, and Controlled Study; and (3) Source Type: Journals; (4) Subject Area: Biochemistry, Genetics and Molecular Biology.

### Study Selection Process

**Figure 1:**
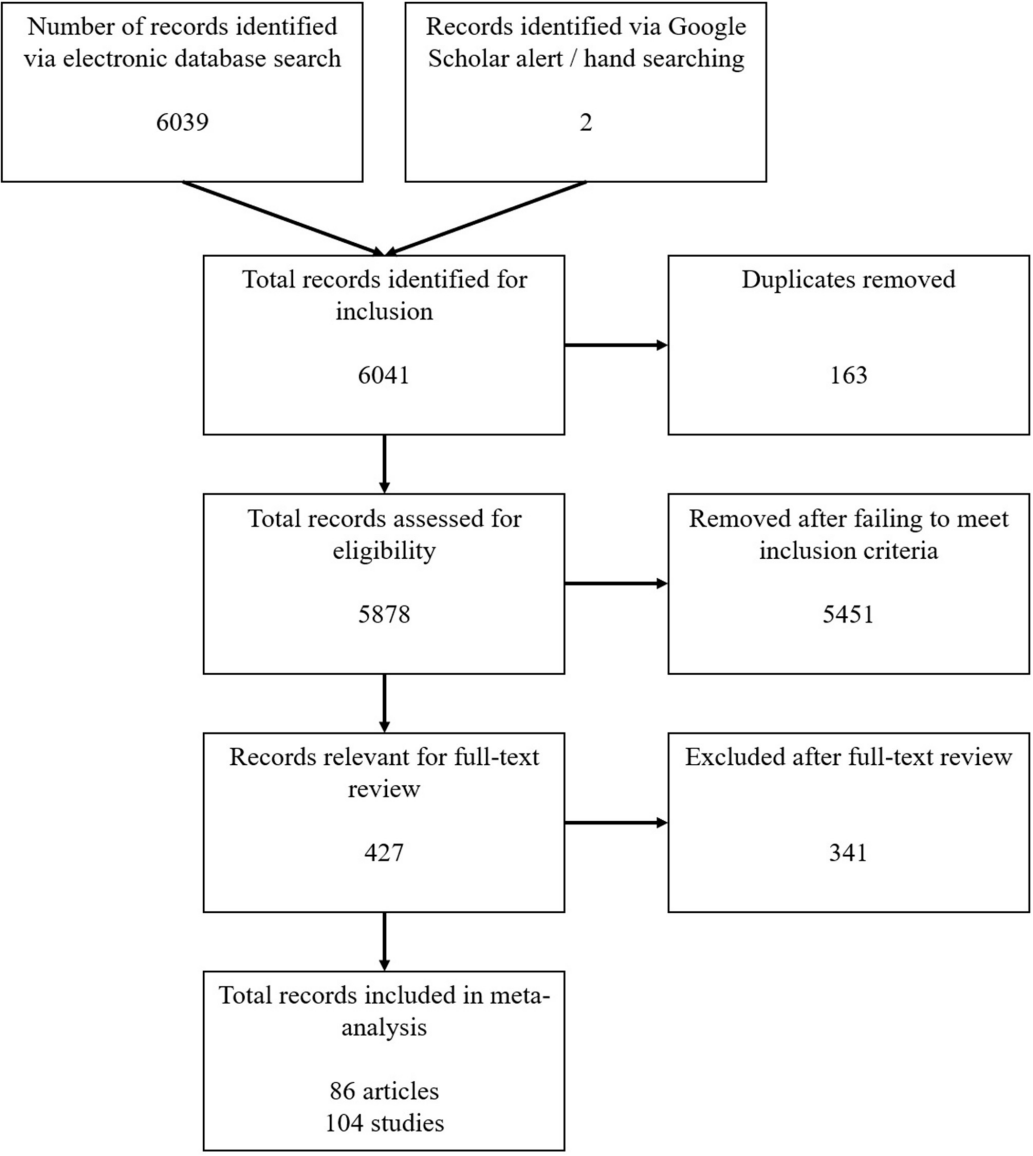
Flow-chart for inclusion and exclusion of articles in the meta-analysis.

In five steps, we identified the total number of included studies: 86 articles, comprising 104 studies, ranging from 1992 to 2016. These steps are presented graphically in Figure 1.

1. The total number of records identified via electronic database searching was 6,039.
2. Additional records were identified via Google Scholar alert (*n* = 1) and hand searching (*n* = 1). Therefore, the total number of records were 6,041.
3. Duplicate records were identified (*n* = 163) and removed, leaving 5,878 records.
4. The title and abstracts of the remaining records (*n* = 5,878) were reviewed, based on the four selection criteria. Ninety percent of the records were independently assessed by two reviewers. The reviewers disagreed about the inclusion of 16% of the records, which often resulted from different interpretations about whether they met inclusion criteria, for example, whether the behaviour in question was a PEB, or whether the independent variable was indeed a measure of identity. Discussion resolved differences to reach a consensus, which occurred during all stages of the double-reviewing process. Based on this step, 5,451 records were excluded, leaving 427 records for full-text review.
5. Full-text articles (*n* = 427) were reviewed based on the four selection criteria. Fifty percent went through the above double-blind review process. The reviewers disagreed about the inclusion of 9% of the articles. After this step, 341 articles were excluded, leaving a total number of 86 articles including a total number of 104 studies for the meta-analysis.

In Step 4 of the study selection process, the main cause of disagreement was due to papers not clearly using the word identity, or PEB in the abstract. Although the definitions of PEB and identity were agreed upon at the start and did not change by the end of meta-analysis, the omissions and inconsistencies of the term identity or PEB meant Reviewer 1 was more likely to include papers when (synonyms for) identity and PEB were not included in the abstract (i.e., Eligibility Criterion 1) compared to Reviewer 2. Through discussions, the two reviewers agreed on the interpretations of these abstracts, and that to meet the Eligibility Criterion 1, identity and PEB needed to be explicit and not ambiguous for inclusion for the next Step 5.

In Step 5 of the study selection process, the main difference occurred because often the papers did not clearly define identity and used many different terms to refer to the same identity construct within the paper (Udall et al., 2020). Again, Reviewer 1 was more likely to include papers when (interpretations for) identity and PEB were not included in the abstract (i.e., Eligibility Criterion 1) compared to Reviewer 2. Again, through discussions, the two reviewers noticed the disagreements were again regarding the ambiguity of identity and PEB relating to Eligibility Criterion 1. The reviewers came to an agreement to exclude those papers that did not explicitly look at identity or PEB in line with Eligibility Criterion 1.

### Data Extraction Process and Analytical Procedures

The extracted data included article information, study number, studied identity type, studied PEB measure (self-reported, observed, or both), sample size, research design (laboratory/field), sample type (student, non-student, or both), overall effect size (unweighted), weighted effect size, sampling variance, *z*-score, lower confidence interval (CI), and upper CI.

In line with previous research (Abrahamse and Steg, 2013), we converted all effect sizes, where necessary to Pearson's *r* for consistency and comparability purposes (Pearson, 1895). The studies sometimes included multiple PEBs or identities, analysed with multiple regression on the same participants. If we had included the coefficients for each identity-PEB relationship, we would have included the same participants multiple times which breaks the assumptions of a random effects meta-regression analysis. Therefore, when studies included multiple measures of identity or PEB, average correlation coefficients were calculated to avoid double counting of participants enabling us to be in line with the assumptions of a random effects meta-analysis.

The data was analysed using the R metafor package (Viechtbauer, 2010). We used a random effects meta-regression model to account for both within—(sampling error) and between-study variance (Overton, 1998). We calculated the mean Pearson's *r* of all identities on PEB by calculating the mean *r*, weighted by the sample sizes of each study (Hunter and Schmidt, 2004). To assess if the weighted Pearson's *r* differed significantly, we used Fisher's (1925) r-to-z transformation. We will report on the variation between studies, the weighted Pearson's *r* of each study, weighted by the studies' sample size, and include the 95% CI for the Pearson's *r* estimate. We will also indicate if the Pearson's *r* are considered small (*r* ≤ −0.10/*r* ≥ 0.10), medium (*r* ≤ −0.30/*r* ≥ 0.30), or large, (*r* ≤ −0.50/*r* ≥ 0.50; Cohen, 1992), and the τ^2^, defined as the variance of the true Pearson's *r* (Borenstein et al., 2011).

To assess the overall presence or absence of heterogeneity among a set of studies we used the Q statistic, and to assess the degree of heterogeneity we used the *I*^2^ statistics and τ^2^ (Higgins and Green, 2011). A percentage below 40 indicates there are no heterogeneity issues in the meta-analysis (Higgins et al., 2003). Furthermore, we used three types of analysis to test for publication bias. Firstly, we checked for funnel plot asymmetry and assessed Egger's regression intercept analysis (Egger et al., 1997). This analysis plots each study's Pearson's *r* against its standard error. In the absence of publication bias, this distribution should be symmetrical around the mean Pearson's *r* (Borenstein, 2005). Secondly, we conducted a trim and fill analysis, which removes smaller studies which may be causing funnel plot asymmetry (Duval and Tweedie, 2000). The analysis iteratively re-computes the mean Pearson's *r*, until the Pearson's *r* is symmetrically distributed. An adjusted Pearson's *r* is then calculated, accounting for publication bias. Finally, we calculated Rosenthal's fail-safe N (Rosenthal, 1979). If the fail-safe N is larger than the number of studies used in the meta-analysis, it is a good sign that there is no publication bias, because one would need many studies to reduce the Pearson's *r*.

Finally, we conducted a meta-regression to assess if moderators alter the identity-behaviour associations. Specifically, a random-effects meta-analysis was conducted with identity, behaviour type (self-report or observed), and research design (laboratory/field), and sample type (student or non-student). Furthermore, the heterogeneity accounted for by the moderators was identified (*R*^2^).

## Results

Over 99 different identities have been studied in a PEB context (Udall et al., 2020). We coded identity as individual (1) or group (2) focused according to how they best fit the definitions described in the introduction. If the identity could be described as individual and group, we coded it as both (0). We coded PEB types as individual (1) or group (2) according to how they fit the definitions in the introduction. Behaviours were categorised as both (0) if it could be described as both individual and group focused. We coded measurement type as PEB self-report (1), PEB observed (2), or both (0). We coded research setting as lab (1), field (2) or unknown (0). We coded sample types as student (1), non-student (2), or both (0). Furthermore, a summary of how many of the included studies were categorised for each moderator can be found in Table 2. In our analysis, we categorised behaviours as whether they are done on their own (e.g., taking our recycling), or are a behaviour that requires being part of group (e.g., attending an activists group meeting). While we did not assess inter-rater reliability, we coded the identities/behaviours through a reading of the measures used in the primary data, and discussion amongst collaborators in cases where there was ambiguity according to how they best fit the definitions of individual/group identities/behaviours. In most cases, it was clear from the primary data whether the behaviour could be performed alone or as part of a group.

**Table 2:** Summary of individual and group identities included for analysing type of behaviour, measure, research setting, and sample types.

		**Individual identities (*n* = 58)**	**Group identities (*n* = 29)**	**Individual PEB (*n* = 72)**	**Group PEB (*n* = 14)**
Type of PEB	Individual	46	16	–	–
	Group	3	8	–	–
	Both	9	5	–	–
Type of identity	Individual	–	–	46	3
	Group	–	–	16	8
	Both	–	–	10	3
Type of PEB measure	Self-report	57	26	70	12
	Observed	1	2	0	2
	Both	0	1	20	0
Research setting	Laboratory	9	6	12	2
	Field	46	23	58	11
	Information unavailable	3	0	2	1
Sample type	Student	17	7	17	4
	Non-student	36	21	32	10
	Both	5	1	6	0

In a series of nine different meta-regression models, the associations between the type of identity and PEB in question are given along with the moderating results—how the moderators contrast from the conditions labelled zero (0). All results reported are given as a Pearson's *r*. In each of the nine random effects meta-regressions, the moderator Pearson's *r* is interpreted as the difference between the moderated Pearson's *r* and the (unmoderated) main association for that model. Each reported moderator Pearson's *r* is the relative difference to the unmoderated (no moderation) Pearson's *r* for that model. Therefore, the Pearson's *r* for a moderated effect should be calculated by adding the unmoderated (no moderation) and moderated Pearson's *r* values.

### Aim 1: Identity and PEB

**Figure 2:**
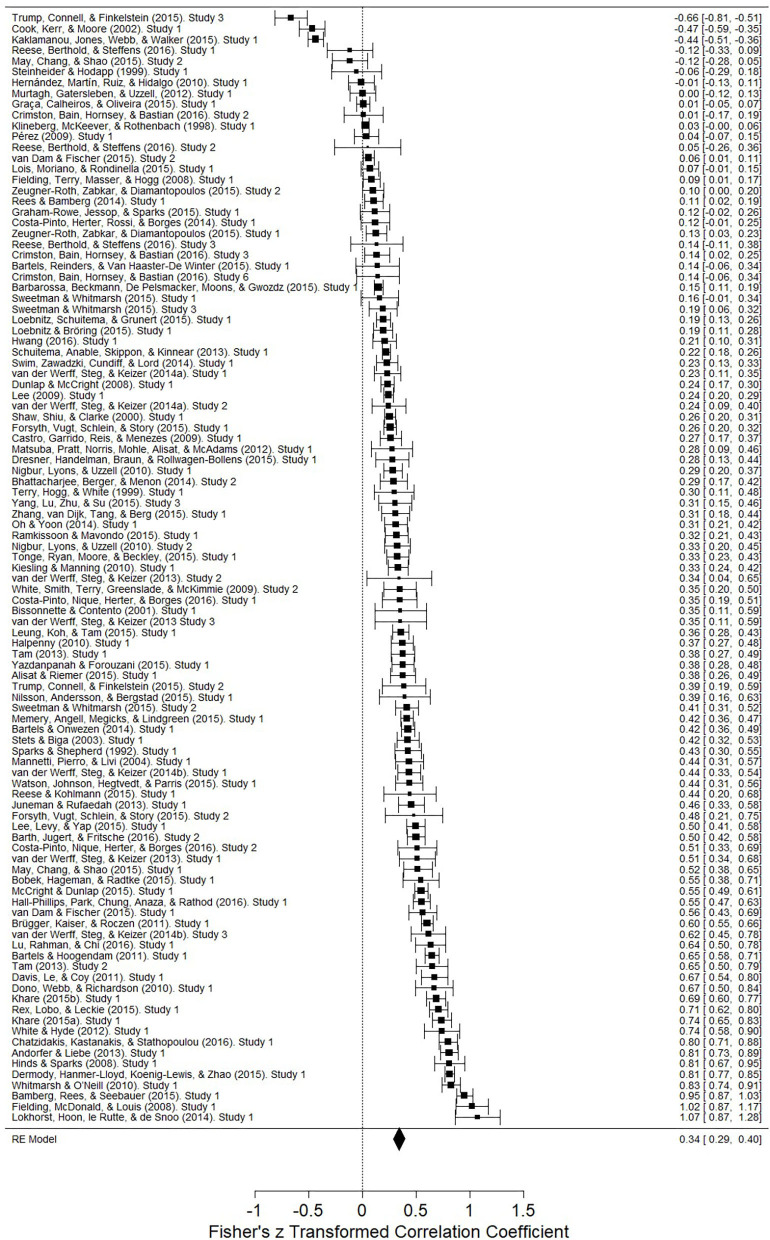
Forest plot of Pearson's *r* regression coefficient, associated 95% confidence intervals (CI) for studies in a random-effects meta-analysis regression.

**Figure 3:**
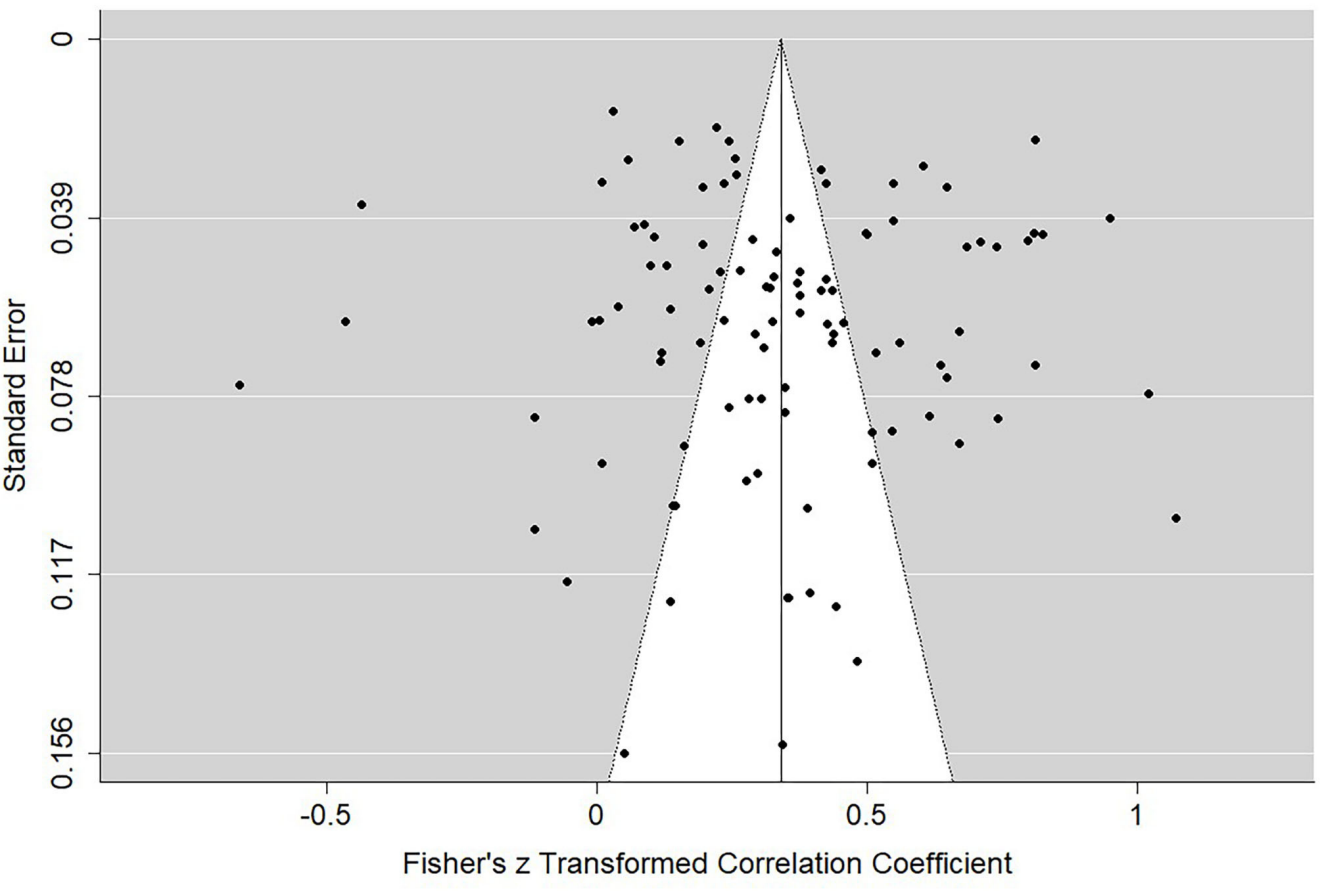
Funnel plot of study residuals against standard error for the model comparing all identity's on all PEBs without moderators.

We meta-analytically looked at the 99 different identities that were identified in a PEB context, for example, pro-environmental identity (Kaklamanou et al., 2015), moral identification (May et al., 2015), and place identity (Hernández et al., 2010). We were interested to capture all the identities, regardless of how they were defined and how they were studied (e.g., experimental, correlational) to identify whether, overall, the identities associated with PEB. A list of all the different identity-PEB associations are in Supplementary Table 1. Figure 2 shows the forest plot with the weighted Pearson's *r* for each study, the overall weighted Pearson's *r*, and accompanying statistics, under the random effects meta-regression model. Overall, identity associated with PEB as shown in Table 3 and the weighted average Pearson's *r* was medium (*r* = 0.340, *p* < 0.001). Our analyses indicated no evidence of publication bias (Table 4). To visually assess publication bias, see the funnel plot in Figure 3. The funnel plot shows the Pearson's *r* estimate of each study against the standard error of each study. Studies with a more reliable estimate will have lower standard error, and therefore should appear near the top-centre. As more rigorous studies to have a lower standard error, but also more conservative estimate of the Pearson's *r*, we should expect to see a symmetrical inverted V-shaped distribution. We found relatively many highly reliable studies represented by low standard errors, and there was symmetrical distribution of the Pearson's *r* estimates around the mean. This finding explains why the Egger's regression intercept analysis showed no indication for publication bias (*Z* = −0.531, *p* = 0.595). The trim and fill analysis showed that zero studies were trimmed. Finally, the Rosenthal's fail-safe N analysis revealed that 167,406 studies with Pearson's *r* of zero would be needed to render the Pearson's *r* non-significant. Therefore, we conclude that identities are positively and significantly associated with PEB.

**Table 3:** Moderator analyses of studies using identities in PEB.

**Meta-regression models**	**Moderators**	**Sample size**	**Pearson's *r* (unweighted)**	**Pearson's r (weighted)**	**Sampling variance**	**Z-Score**	**Lower C.I**.	**Upper C. I**.	***p***
Model 1: All identities and all PEB	No moderators	49,860	0.308	**0.340**	**0.005**	**12.155**	**0.285**	**0.395**	**< 0.001**
	Identities: Individual			0.058	0.078	0.741	−0.095	0.211	0.459
	Identities: Group			−0.042	0.089	−0.475	−0.217	0.132	0.635
	PEB: Individual			0.007	0.073	0.102	−0.136	0.151	0.919
	PEB: Group			0.023	0.101	0.226	−0.175	0.221	0.821
	PEB: Self-report			−0.104	0.213	−0.489	−0.521	0.131	0.625
	PEB: Observed			−0.397	0.278	−1.43	−0.941	0.147	0.153
	Research setting: Lab			0.163	0.168	0.970	−0.166	0.492	0.332
	Research setting: Field			0.248	0.168	1.477	−0.081	0.578	0.140
	Sample: Student			0.155	0.123	1.256	−0.087	0.396	0.209
	Sample: Non-student			−0.060	0.110	−0.551	−0.276	0.155	0.582
Model 2: All identities and individual PEB	No moderators	36,038	0.311	**0.340**	**0.034**	**10.090**	**0.274**	**0.406**	**< 0.001**
	Identities: Individual			0.123	0.102	1.205	−0.077	0.324	0.228
	Identities: Group			−0.007	0.120	−0.054	−0.242	0.229	0.957
	PEB: Self-report			−0.151	0.221	−0.685	−0.583	0.281	0.493
	PEB: Observed			−0.447	0.352	−1.270	−1.135	0.242	0.204
	Research setting: Lab			0.230	0.182	1.265	−0.127	0.587	0.206
	Research setting: Field			0.176	0.185	0.948	−0.187	0.538	0.343
	Sample: Student			0.047	0.166	0.280	−0.290	0.372	0.780
	Sample: Non-student			−0.084	0.147	−0.572	−0.373	0.205	0.567
Model 3: All identities and group PEB	No moderators	4,522	0.277	**0.328**	**0.081**	**4.044**	**0.169**	**0.487**	**< 0.001**
	Identities: Individual			−0.071	0.116	−0.610	−0.298	0.156	0.542
	Identities: Group			−0.071	0.098	−0.722	−0.264	0.122	0.471
	PEB: Self-report			0.045	0.220	0.204	−0.387	0.476	0.839
	Research setting: Lab			**−0.806**	**0.199**	**−4.043**	**−1.197**	**−0.415**	**< 0.001**
	Sample: Student			**0.583**	**0.134**	**4.341**	**0.320**	**0.847**	**< 0.001**
	Sample: Non-student			−0.168	0.105	−1.604	−0.374	0.037	0.109
Model 4: Individual identities and all PEB	No moderators	33,371	0.336	**0.373**	**0.038**	**9.699**	**0.297**	**0.448**	**< 0.001**
	PEB: Individual			0.024	0.104	0.233	−0.179	0.227	0.816
	PEB: Group			−0.154	0.175	−0.879	−0.496	0.189	0.380
	PEB: Self-report			0.308	0.276	1.118	−0.232	0.848	0.264
	Research setting: Lab			0.248	0.192	1.294	−0.128	0.623	0.196
	Research setting: Field			0.184	0.193	0.955	−0.194	0.562	0.340
	Sample: Student			0.093	0.184	0.502	−0.269	0.454	0.615
	Sample: Non-student			< 0.001	0.174	< 0.001	−0.341	0.341	1.00
Model 5: Individual identities and individual PEB	No moderators	26,041	0.343	**0.381**	**0.044**	**8.632**	**0.295**	**0.468**	**< 0.001**
	PEB: Self-report			0.301	0.282	1.069	−0.251	0.853	0.285
	Research setting: Lab			0.327	0.203	1.608	−0.072	0.725	0.108
	Research setting: Field			0.162	0.211	0.769	−0.251	0.576	0.442
	Sample: Student			0.215	0.222	0.971	−0.219	0.650	0.332
	Sample: Non-student			0.137	0.217	0.631	−0.289	0.563	0.528
Model 6: Individual identities and group PEB	No moderators	869	0.217	**0.247**	**0.047**	**5.300**	**0.156**	**0.339**	**< 0.001**
	Research setting: Lab			**−0.233**	**0.118**	**−1.966**	**−0.465**	**−0.001**	**0.049**
	Sample: Non-student			**−0.167**	**0.075**	**−2.217**	**−0.314**	**−0.019**	**0.027**
Model 7: Group identities and all PEB	No moderators	11,591	0.249	**0.274**	**0.052**	**5.288**	**0.172**	**0.375**	**< 0.001**
	PEB: Individual			0.024	0.115	0.205	−0.202	0.249	0.838
	PEB: Group			0.100	0.138	0.721	−0.171	0.370	0.471
	PEB: Self-report			0.225	0.279	0.808	−0.321	0.772	0.419
	Sample: Student			**0.332**	**0.656**	**2.000**	**0.007**	**0.657**	**0.046**
	Sample: Non-student			−0.148	0.119	−1.249	−0.381	0.084	0.212
Model 8: Group identities and PEB	No moderators	6,880	0.238	**0.249**	**0.067**	**3.724**	**0.118**	**0.381**	**< 0.001**
	PEB: Self-report			−0.053	0.379	−0.140	−0.796	0.690	0.888
	Sample: Student			−0.080	0.317	−0.252	−0.701	0.541	0.801
	Sample: Non-student			−0.285	0.192	−1.490	−0.661	0.090	0.136
Model 9: Group identities and group PEB	No moderators	2,702	0.265	**0.316**	**0.120**	**2.640**	**0.081**	**0.551**	**0.008**
	PEB: Self-report			**0.849**	**0.124**	**6.867**	**0.607**	**1.091**	**< 0.001**
	Sample: Student			**0.593**	**0.094**	**6.306**	**0.408**	**0.777**	**< 0.001**
	Sample: Non-student			−0.140	0.084	−1.667	−0.304	0.025	0.096

*C.I., Confidence intervals; Bold text, significant results*.

**Table 4:** Publication bias check of studies using identity types in PEB.

**Type of identity**	**Type of PEB**	**Egger's regression intercept analysis**	**Trim and fill analysis *(Estimated # of missing studies on right side)***	**Rosenthal's fail safe *N***
All	All	Z = −0.531, *p* = 0.595	0 (S.E. = 5.956)	167,406, *p* < 0.001
	Individual	Z = 0.817, *p* = 0.414	21 (S.E. = 5.430)	84,738, *p* < 0.001
	Group	Z = 1.723, *p* = 0.805	3 (S.E. = 2.553)	2,660, *p* < 0.001
Individual	All	Z = 0.620, *p* = 0.535	0 (S.E. = 4.507)	67,294, *p* < 0.001
	Individual	Z = 1.224, *p* = 0.221	0 (S.E. = 4.036)	44,492, *p* < 0.001
	Group	Z = −1.371, *p* = 0.170	2 (S.E. = 1.514)	64, *p* < 0.001
Group	All	Z = −1.793, *p* = 0.073	0 (S.E. = 3.163)	8,523, *p* < 0.001
	Individual	Z = −0.175, *p* = 0.861	0 (S.E. = 2.027)	2,457, *p* < 0.001
	Group	Z = −1.376, *p* = 0.169	2 (S.E. = 1.862)	775, *p* < 0.001

### Aim 2: Individual and Group Identities and PEB

To test the unique contribution of individual and group identities on PEB, we categorised the identities as either individual, group, or both. For individual identities (Table 2, *n* = 58), we found a medium weighted average Pearson's *r* (*r* = 0.373, *p* < 0.001). As shown in Table 4, Egger's regression intercept analysis (*Z* = 0.620, *p* = 0.535), the trim and fill analysis (zero studies were trimmed), and, the Rosenthal's fail safe N (67,294 studies with Pearson's *r* of zero would be needed to render the Pearson's *r* non-significant) showed no indication of a publication bias. For group identities (Table 2, *n* = 29), we found a small, but close to medium Pearson's *r* (*r* = 0.274; *p* < 0.001; Table 3). Table 4 shows that there was no publication bias: Egger's regression intercept analysis (*Z* = −1.793, *p* = 0.073), the trim and fill analysis (zero studies were trimmed), and the Rosenthal's fail-safe N was 8,523 studies.

Next we explored whether individual and group identities related differently to PEB. The moderation analyses (Table 3) showed that neither the Pearson's *r* estimate for individual identities on PEB (*r* = 0.058, *p* = 0.459), nor for group identities on PEB (*r* = −0.042, *p* = 0.635), were significantly different from the overall Pearson's *r* of identity on PEB. However, these results are on a (smaller) study level only (*n*_study max_ = 104). Therefore, we complemented these moderation tests with a Fisher's r-to-z transformation to compare the two regressions reported above (i.e., on the larger sample level rather than the smaller study level). This showed that the two Pearson's *r* were significantly different (*r*_1_ = 0.373, *n*_1_ = 33,371, *r*_2_ = 0.274, *n*_2_ = 11,591; *z* = 12.95, *p* < 0.001). Therefore, individual identities associate with PEB more so than group identities.

### Aim 3: Identity and Individual and Group PEBs

Firstly, we explored whether all 99 identities associated with individual PEB. We focused only on studies with individual PEB (Table 2, *n* = 72). As Table 3 indicates, we found the weighted average Pearson's *r* was medium (*r* = 0.340; *p* < 0.001). There was no publication bias (Table 4), as shown by Egger's regression intercept analysis (*Z* = 0.817, *p* = 0.414), the trim and fill analysis (a total of 21 studies were trimmed), and, the Rosenthal's fail safe N (67,294 studies with Pearson's *r* of zero would be needed to render the Pearson's *r* non-significant). Therefore, identities are associated with individual PEB.

Secondly, we explored if all identities associated with group PEB. We focused only on studies with group PEB (Table 2, *n* = 14). As Table 3 indicates an association with a medium weighted average Pearson's *r* (*r* = 0.328; *p* < 0.001). As Table 4 shows, Egger's regression intercept analysis revealed that there was no publication bias (*Z* = −1.723, *p* = 0.805). The trim and fill analysis (a total of 3 studies were trimmed), and the Rosenthal's fail-safe N was 84,738 studies. Therefore, identities are associated with group PEB.

To complement the analyses above, we explored whether all identities related differently to individual and group PEB. The moderation analyses showed that neither the Pearson's *r* estimate for identities on individual PEB (*r* = 0.007, *p* = 0.919), nor for identities on group PEB (*r* = 0.023, *p* = 0.821), was significantly different from the overall Pearson's *r* of identity on PEB. To supplement these moderation tests, we also performed Fisher's r-to-z transformation to compare the two regressions reported above. This showed that the two Pearson's *r* were not significantly different (*r*_1_ = 0.340, *n*_1_ = 36,038, *r*_2_ = 0.328, *n*_2_ = 4,522; *z* = 0.86, n.s.). Therefore, identities are not differently associated with individual or group PEB.

To explore the extent to which individual and group identities match individual and group PEB, we first looked at the observed Pearson's *r* in Table 3 when identity and PEB associations were matched. When identity and PEB associations were individually matched (individual identity-individual PEB), the weighted Pearson's *r* was *r* = 0.381; and, when they were group matched (group identity-group PEB) the weighted Pearson's *r* was *r* = 0.316. The individually matched *r* was 0.041 more than the average observed *r*. The group matched *r* was 0.024 less than the average observed *r* given the average observed *r*. Fisher's r-to-z transformation showed that the individual-matched association was significantly different from the average (*r*_1_ = 0.381, *n*_1_ = 26,041, *r*_2_ = 0.340, *n*_2_ = 49,860; *z* = 6.16, *p* < 0.001), yet the group-matched association was not (*r*_1_ = 0.316, *n*_1_ = 2,702, *r*_2_ = 0.340, *n*_2_ = 49,860; *z* = −1.36, n.s.).

Next, we looked at the observed Pearson's *r* in Table 3 when identity and PEB associations were mismatched. We found that individual identity and group PEB associations were *r* = 0.247; group identity and individual PEB associations were *r* = 0.249. The individual identity-group PEB *r* was 0.093 less than the average observed *r*. The group-identity-individual PEB *r* was 0.091 less than the average observed *r*. Fisher's r-to-z transformation showed that these mismatched associations significantly differed from the average, respectively (*r*_1_ = 0.247, *n*_1_ = 869, *r*_2_ = 0.340, *n*_2_ = 49,860; *z* = −2.97, *p* < 0.01) and (*r*_1_ = 0.249, *n*_1_ = 6,880, *r*_2_ = 0.340, *n*_2_ = 49,860; *z* = −7.75, *p* < 0.001).

Lastly, we also assessed if the matched category of identity-PEB estimates significantly differed from the mismatched identity-PEB associations. The Pearson's *r* estimate for the highest scoring matched association (i.e., individual identity and individual PEB) was higher than the estimate for the lowest scoring mismatched associations (i.e., individual identity and group PEB), while the difference between the lowest scoring matched associations and highest scoring mismatched associations were lower. Fisher's r-to-z transformation showed that these were significantly different, respectively (*r*_1_ = 0.381, *n*_1_ = 26,041, *r*_2_ = 0.247, *n*_2_ = 869; *z* = 4.31, *p* < 0.001) and (*r*_1_ = 0.316, *n*_1_ = 2,702, *r*_2_ = 0.249, *n*_2_ = 6,880; *z* = 3.21, *p* < 0.01). Based on these findings, we conclude categorising identity and PEB as individual or group is useful because individual identity better explains individual PEBs and group identities better explain group PEBs.

### Aims 4-6: Measures, Research Setting, and Samples as Moderators

In the final part of our meta-analysis, we explored the extent to which the behavioural measurement (self-reported vs. observed), research setting (laboratory vs. field), and sample type (student vs. non-student) moderated the identity-PEB associations. The associations between all identity types and all PEB types were not moderated by the type of behavioural measure (self-report: *r* = −0.104, *p* = 0.625 vs. observed: *r* = −0.397, *p* = 0.153). However, a more detailed analysis of individual vs. group identity and PEB revealed that the association between group identity and group PEB was significantly moderated by self-reports (*r* = 0.849, *p* < 0.001). This finding indicates that the associations for group identity-group PEB were stronger for self-reported behaviours than observed behaviours. Thus, to some extent the type of behavioural measurement alters the associations between identity and PEB.

The associations between all identity types and all PEB types were not significantly moderated by the laboratory setting (*r* = 0.163, *p* = 0.332) or field setting (*r* = 0.248, *p* = 0.140). However, the more detailed analysis showed that all identity types combined with group PEB were significantly moderated by the laboratory research setting (*r* = −0.806, *p* < 0.001) indicating that the associations were weaker in a lab than in a field setting. Also, the associations between individual identities and group PEB were significantly moderated by the laboratory research setting (*r* = −0.233, *p* < 0.05), indicating that the associations decreased more in a lab than in a field setting. Hence, to some extent the research setting alters the associations between identity and PEB.

The sample type (student or non-student) might alter the associations between identity and PEB. The associations between all identity types and all PEB types were not significantly moderated by student samples (*r* = 0.155, *p* = 0.209) or non-student samples (*r* = −0.060, *p* = 0.582). However, the identity-group PEB associations were larger in student samples (*r* = 0.583, *p* < 0.001). Yet, the group identity-overall PEB associations were smaller in student samples (*r* = −0.332, *p* < 0.05). Finally, the individual identity-group PEB associations were weaker in student samples (*r* = −0.167, *p* < 0.05). Thus, to some extent the sample type (student or non-student) altered the associations between identity and PEB.

## Discussion

To follow the core structure of this article, the findings and implications for each of the six aims will be discussed in their respective order. Then we will address possible future research directions as well as practical implications, before ending with overall conclusions.

### Aim 1: Overall Identity and Behaviour

Our random-effects meta-analysis showed that identity associates with PEB with a medium Pearson's *r*. These findings are in line with the two key theories of identity, identity theory (Stryker and Burke, 2000) and social identity theory (Tajfel and Turner, 1979), as well as with the more recently proposed PEB-identity theory (Udall et al., 2020). These theories assume that identity is a key antecedent of (pro-environmental) behaviour. Hence, the four dominant psychological theories used in PEB research (Klöckner, 2013), namely theory of planned behaviour (Ajzen, 1991), norm-activation theory (Schwartz, 1992), value-belief-norm theory (Stern, 2000), and habits (Verplanken and Aarts, 1999) might be significantly improved by including the concept of identity. Our results showed that—overall—there is an association between identity and PEB. Yet, our research also shows that not all the identities need to be included as we found different associations for different types of identity and PEB.

### Aim 2: Different Identities and Overall Behaviour

Based on self-categorisation theory (Turner et al., 1987) we categorised all these identities in two main ways, either as an individual identity or a group identity. Such categorisation is sometimes done in the primary articles but is often implicitly assumed. Our research reveals that regardless of what identity we use (individual or group), they relate to PEB. However, we showed that individual identities-overall PEB associations were stronger when compared to group identities-overall PEBs associations. This finding suggests it is useful to categorise identities as individual or group because the two groups have different association strengths.

We found that group identity-PEB associations were overall weaker than the individual identity-PEB associations. For example, one study (Fielding et al., 2008a) investigated the relationship between an individual identity (self-identity with environmental activism) and a group identity (social identity: group membership of an environmental group) with intention to engage in environmental activism. While researchers found that both were significantly associated with activism, the group identity had a smaller Pearson's *r* than the individual identity. A weaker association for the group identity might be because these identities need the presence of the group to strengthen the identity, thus association with PEB, while the individual identity does not need this cue. We found that in many studies the group was not physically present when these group identities were measured (e.g., Dunlap and McCright, 2008) or cued (made salient) experimentally (e.g., Costa-Pinto et al., 2014, 2016). Behaviour might occur because of group identity if the relevant group is physically present because this functions as a suitable cue (makes salient) the group identity. Perhaps merely thinking about the physical presence of a group (e.g., Costa-Pinto et al., 2014, 2016) may not function sufficiently as a cue (make salient) the group identity and thus not lead to the corresponding behaviours. Perhaps group identities are reliant on the group in line with social identity theory (Tajfel and Turner, 1979). Therefore, we suggest that when research is focused on a particular in-group context, or, when the context is relying on group cues, it might be necessary for future researchers to explicitly focus on group rather than individual identities and account for this missing and weak group setting. In contrast, focus on the individual identities when the group settings are absent.

### Aim 3: Identity and Different PEBs

As we distinguished between individual and group identities, we also categorised PEBs accordingly (Tajfel, 1982). Overall identities related to individual and group PEBs. However, individual identities associated more strongly with individual PEBs while group identities associated more strongly with group PEBs. These finding suggest that future research might benefit from distinguishing and matching these identities and PEBs as individual or group. This matching is in line with social identity theory (Tajfel, 1982; Stern, 2000) where the associations between identity and PEB are assumed to be particularly strong when the identity and PEB are matched and weakest when they are mismatched. However, future research is needed because scholars have not explicitly categorised and assessed identities and PEBs accordingly. At a minimum, we suggest being explicit about the choice of individual-/group-focused identity and/or individual-/group-focused PEB. Finally, and ideally measuring these combinations would shed the light on the matching proposition. For example, we suggest measuring an individual identity (e.g., self-identity with environmental activism) and a group identity (e.g., social identity: group membership of an environmental group) in relation to an individual PEB (e.g., PEB at home) and a group PEB (e.g., intention to engage in environmental activism with peers).

### Aim 4-6: Measures, Research Designs, and Samples

Finally, our results indicate that the methods used in identity and PEB research moderate the identity-PEB associations. We focused on three moderators, specifically, the types of behaviour measures, research setting, and sample type (Wood et al., 2015). Firstly, the findings indicated that self-report vs. objective measures moderated identity and PEB associations. Specifically, we revealed group identities-group PEB associations were stronger for self-reports. A reason for this result could be that people wish to feel, experience, and act in ways that are consistent with the group identity (Tajfel and Turner, 1979). Consequently, group identities will guide self-reports of group behaviours, because it is easier to report than to execute the behaviour.

Secondly, our findings show that studies conducted within the lab, rather than the field, negatively moderated certain identity-PEB associations (i.e., all identities-group PEB; individual identities-group PEB). In field settings these associations were found to be larger, possibly because of more researcher degrees of freedom—The inherent flexibility regarding how studies are designed and analysed (Simmons et al., 2011). Also, as recently suggested (e.g., Udall et al., 2020), when in the field, factors which may be more likely to cue a person's identity, may be more salient, such as promotional messages like the “Veganuary” campaign in the United Kingdom that provides information on posters, television adverts, and in supermarkets to encourage a meat and dairy free (Vegan) January. In the field vs. the lab there is less control over extraneous variables that might act as situational cues that make salient the identities unintentionally. These cues change aspects of the environment that might affect the participant's identity and subsequent PEB. These extraneous and situational cues may be less prevalent in a lab because they can be more easily controlled for and minimised.

Furthermore, weaker associations (all identities-group PEB; individual identities-group PEB) in the lab contradicted with the popular “demand effects hypothesis” (Haney et al., 1973; Wood et al., 2015). This hypothesis assumes associations will be stronger in laboratory rather than field settings because of demand characteristics. Participants may feel pressured to act consistently with their perceived expectations of the researcher. However, as we revealed the opposite, social identity theory (Tajfel and Turner, 1979) might shed some light on these findings. Social identity theory assumes that people carry out behaviours because of their group's expectations, irrespective of whether the group is present. However, given our findings, it might be that perhaps the group pressure needs to be salient, at least to some extent, for the behaviour to occur. In labs, identity may relate to group PEB to a lesser extent due to lower group-based demand characteristics. The participants do not feel pressure to act consistently with their intentions or self-reports because the group presence is often missing. However, our research also revealed that group PEBs are less well-studied. Further research is thus needed to determine this boundary condition for group PEBs. We have the above theorising as a starting point for future research. We suggest focusing on measuring the same identity and group PEB behaviour in the lab and field using the same sample, to further understand these associations.

Finally, sample type moderated some identity-PEB (i.e., all identities-group PEB; Individual identity-group PEB; Group identity-group PEB) associations. Indeed, reliance on student samples has largely been criticised in psychological research with the argument that they do not reflect the population at large (Henrich et al., 2010). Critics point out that the greater the student composition of the sample, the larger the associations evidenced (Henrich et al., 2010; Wood et al., 2015). Students are assumed to be more susceptible to cognitive dissonance, show increased levels of self-monitoring, greater inclination toward attitude change, and, are more susceptible to group presence compared to others (Wood et al., 2015). In line with these critiques, our findings showed that student samples moderated all identity-group PEB, and group identities-all PEB, compared to non-student samples. Furthermore, non-student samples negatively moderated individual identity-group PEB associations. Future research might benefit from these insights, by carefully considering the sample used based on which identity-PEB association is examined. We argue that this approach is especially needed when focusing on group identities and group PEBs. For example, if the aim is to understand students, then this sample type is suitable in this context. However, if the aim is to understand the population in general, then future studies should seek to use a representative sample.

## Future Directions

While our paper focused on identifying whether the different identities studied in a PEB context associated with PEB, we acknowledge that future research needs to focus on why these associations are expected. For example, identity may associate with PEB via norms (Tajfel and Turner, 1979). We lay the foundations for much more specific and detailed future work as our study revealed and summarised what identities were studied, what measures were used, and in what settings. With this “clean up” and overview, scholars can now further explore the mechanisms by which identity associates with PEB. For example, one mechanism might be schemas: internally stored information about situations and expected behaviours (Markus, 1977; Stryker and Burke, 2000). The second mechanism might be norms: a salient group identity activates a group norm, which encourages the PEB. Social norm research proposes that individuals have multiple social norms which must be salient or activated to affect PEB (Cialdini et al., 1990). However, our meta-analysis of the existing literature shows that these mechanisms that best explain identity-PEB associations need exploring. While different group identities may associate with PEB via norms, there are three reasons why this is yet to be known. Firstly, it is currently not known which social norms might activate a specific group identity. Secondly, it is not known whether the social norms associated with a particular group include the specific PEB being measured. Thirdly, even if the group identity included social norms and behaviour, it is not known if, when the person was asked, whether the social norm was present. We evoke these explanations, as they may explain why we observed a weaker relationship for group identities than for individual identities. However, future research is necessary to assess the group identities and PEB alongside variables like social norms. Furthermore, while we did not measure inter-rater reliability of our coding of individual/group identity/behaviours, this paper takes a first step to consolidate the topic in such a way. Future research would benefit from more explicitly categorising their measures along these lines to further confirm how these different categories of identity relate to different categories of behaviour.

Furthermore, given the current state of the literature, this research could not examine quasi- experimental (Matsuba et al., 2012) vs. experimental (Van der Werff et al., 2013, 2014a,b; Bhattacharjee et al., 2014; Costa-Pinto et al., 2014, 2016) vs. correlational designs (Murtagh et al., 2012). These designs were “lumped” together (Thomas et al., 2019), which is not uncommon in meta-analyses (Caldwell and Welton, 2016). While we agree it would be beneficial to “split” the data in this way, there are simply not enough (quasi-)experimental primary studies to explore this. For example, 11% of identity-PEB studies used experimental designs and only 14% used within-participant designs (Udall et al., 2020), as such aggregation was required (Caldwell and Welton, 2016). Based on there being scant within-participant experimental (or longitudinal) data in the field, we suggest that it is not possible to make any strong claims about causality of identity and PEB associations. Hence, our research shows this is a much needed area for future research. Also, assessing these associations independently of other possible extraneous variables and situational cues that might be confounding the specific identity-PEB associations is needed. We suggest more research is needed in controlled (laboratory and field) settings using (quasi-)experimental and longitudinal research designs. Specifically, measuring the same PEB as an intention, a self-reported PEB, an observed PEB, an individually focused PEB, and as a group focused PEB, alongside the identity types in question would help researchers to better understand these additional moderators on identity-PEB associations.

Our research also reveals that group identities in general were under-represented and were examined mainly in student samples. Yet we argue that, especially for understanding group PEB, the setting needs to represent reality as much as possible, as the setting itself might raise or lower certain group identities. Hence, to better understand the function of group identities and behaviours, we suggest that future research needs to test identities in these ways alongside factors that may be contributing to the increased identity and behaviour associations (Balakrishnan, 1999; Prince et al., 2008; Henrich et al., 2010; Wood et al., 2015). One approach could be by creating settings that “represent realities,” for example by considering “living labs research” whereby studies are conducted in a residential home research facility where people's behaviour and identity can be observed, non-invasively, and in real time (Schwartz et al., 2015).

Our research was the first to assess identity in the PEB research-field meta-analytically. However, we acknowledge the limitation of when this analysis was conducted. Since our meta-analysis was conducted, there has been new empirical and theoretical work in this field which we now discuss. A few notable examples include the development of an Ecological Identity Scale to measure an “ecological identity” (Walton and Jones, 2017). This identity measure captures both an individually focused and group focused identity. The individually and group focused ecological identity associated directly and indirectly with a wide range of individually focused PEBs (Walton and Jones, 2017). Furthermore, the value-identity-personal norm model has been developed where an individually focused identity (environmental identity) plays a central role for individually focused PEBs and observed individually focused PEB: Participation in smart energy systems (Van der Werff and Steg, 2016). The Social Identity Model of Pro-Environmental Action (SIMPEA; Fritsche et al., 2018) was also introduced which proposes the role of four social identity processes (Fritsche et al., 2018). Specifically, secondary data suggests that group identity, group norms and goals, and collective efficacy determine environmental appraisals, and both individual and group PEBs. These processes are driven by personal and collective emotions and motivations that arise from environmental appraisal and operate on both a deliberate and automatic processing level (Fritsche et al., 2018). Research also suggests interventions targeting environmental identity not only increase the adoption of a first PEB, but also increases the likelihood of adopting another PEB, referred to as positive spillover (Maki et al., 2019). Finally, research has begun to further promote an individually and group focused green identity, using the green identity labelling technique (Schwartz et al., 2020). The green identity labelling technique increased individual PEB purchases online, in a laboratory, and in two field experiments (Schwartz et al., 2020). With our meta-analytical findings, we provide an overview of the field before such recent developments, yet our findings are still relevant so researchers can learn from the past to inform the future. Hence, we hope that scholars working on these recently emerged lines of inquiry also take note of our findings and suggestions.

Furthermore, in our meta-analysis, we assessed study quality and the potential that studies have been selectively published or the “file-draw effect,” by assessing the distribution of the correlation coefficients and sample sizes. We found no risk of bias suggesting there was no large risk of this “file-draw effect.” However, since our meta-analysis, new approaches have also been developed that can be adapted and applied in our PEB research in future to deepen our understanding of the potential risk of bias of each study. Specifically, future research would benefit from assessing the risk of bias within a study using an example like the consensus-based standards for the selection of health measurement instruments (COSMIN) checklist (Mokkink et al., 2018). Furthermore, drawing on past research, study quality can be assessed using a revised tool for the quality assessment of diagnostic accuracy studies (Quadas)-2 (Whiting et al., 2011). Future research would benefit from creating checklists like these that are PEB research appropriate, namely, for correlational, non-intervention, and non-patient research. While we found no risk of bias for overall quality in our sample, future research would benefit from assessing the moderating role of specific individual study biases on the identity-PEB associations. Based on what our meta-analysis reveals and these notable developments in the field, we propose practical implications and policy recommendations.

## Practical Implications

Our meta-analysis reveals which identities might best encourage PEB in future, namely those with a Pearson's *r* and confidence intervals which are above zero as they associate positively with PEB (Supplementary Table 1, Column: 16). Furthermore, in line with the idea that identities guide our thoughts, feelings, and subsequent behaviours (Tajfel and Turner, 1979; Burke, 1980), we found stronger correlations for identities that are conceptually most alike to the theme of PEB (in other words, which were PEB related). Therefore, we propose that identities can be categorised into three categories with respect to how they relate to PEB (Supplementary Table 1, Column 3), namely (1) pro-PEB related (i.e., the identity is actively (positively) related to the PEB definition), (2) anti-PEB related (i.e., the identity is actively appositionally (negatively) related to the PEB definition, and (3) neutral-PEB related (i.e., the identity is neither related nor unrelated, thus is neutral to the PEB definition). Specifically, pro-PEBs best associate with PEBs. Based on the Pearson's *r* and confidence intervals being above zero, focusing on pro-PEB identities and combining this with our dual perspective on individual and group identities, we identify the top 10 individual and group identities for practical interventions, policy makers, as well as for future scholars to target when encouraging PEB in future. In addition, we provide the exact definitions of the identities when these were present in the paper. When these explicit identity definitions were missing, we infer them based on our theoretical understanding of the identity in question.

### Individual Identities

- Self-identity in private nature conservation (Lokhorst et al., 2014): An individual's observations of their own thoughts, feelings, and behaviour that mean they personally prioritise the preservation of the land that they are responsible for personally.
- Self-identity with environmental activism (Fielding et al., 2008a, p. 30): “*Identity (e.g., as an environmental activist) motivates action, and to not engage in role-appropriate behaviour (e.g., environmental activism) may create a state of internal tension due to conflict between identity and actions. In contrast, engaging in role-appropriate behaviour validates individuals' role, and therefore their self-identity*.”
- Behaviour generic self-identity: Pro-environmental (Whitmarsh and O'Neill, 2010): An individual's observations of their own thoughts, feelings, and behaviour that mean they personally prioritise engagement in any PEB.
- Behaviour specific self-identity: Carbon offsetting (Whitmarsh and O'Neill, 2010): An individual's observations of their own thoughts, feelings, and behaviour that mean they personally prioritise a precise PEB—reducing carbon dioxide emissions/other greenhouse gases to compensate for emissions made elsewhere.
- Pro-environmental self-identity (Dermody et al., 2015, p. 1,478): “*Refers to individuals possessing a sense of self that embraces pro-environmental actions*.”
- Environmental identity (Hinds and Sparks, 2008, p. 110): “*Meanings that one attributes to the self as they relate to the environment*.”
- Fair Trade consumer identity (Andorfer and Liebe, 2013): An individual's observations of their own thoughts, feelings, and behaviour that mean they personally prioritise using goods and services that have sustainable and equitable trade relationships with producers of developing countries.
- Internal ethics (Rex et al., 2015; Chatzidakis et al., 2016): “*Refers to individuals' internalised ethical rules and their intrinsic ethical and moral considerations about what is right and wrong and relates to their self-identity*” (Rex et al., 2015, p. 267).
- Self-identity as a recycler (White and Hyde, 2012): An individual's observations of their own thoughts, feelings and behaviour that mean they personally prioritise converting waste into reusable material.
- Green self-identity in environmental protection (Khare, 2015a, Khare, 2015b): An individual's observations of their own thoughts, feelings and behaviour that mean they personally prioritise the preservation of the planet.

### Group Identities

- Social identity: Group membership of an environmental group (Fielding et al., 2008a): An individual's perception that they belong to a group which engages in PEB.
- Group identity with Transition Towns (Bamberg et al., 2015): An individual's perception that they affiliate with people in their municipality that provide PEB initiatives as follows—increase in self-sufficiency and support the reducing of the potential effects of peak oil, climate destruction, and economic instability.
- Group identification with (social) group of environmentalists (Dono et al., 2010): An individual's perception that they belong to a group which engages in PEB.
- Group identification with environmentalist (Dono et al., 2010): An individual's perception that they belong to a group which engages in PEB.
- Social identification with environmentally conscious consumer (Bartels and Hoogendam, 2011; Bartels and Onwezen, 2014): An individual's perception that they belong to a group of customers that use resources in a PEB aware way.
- Social identification with organic consumer (Bartels and Hoogendam, 2011; Bartels and Onwezen, 2014): An individual's perception that they belong to a group of customers that use resources that were grown or raised free of synthetic fertilisers and pesticides.
- Consumer social venture identification (Hall-Phillips et al., 2016, p. 485): “*Defined as the psychological attachment a consumer feels/has to a social venture based on the perceived commonality between their self-concept and a social venture's perceived identity*.”
- Identity similarity with typical recyclers (Mannetti et al., 2004): An individual who perceives themselves as having a high degree of likeness to the ideal group of people that convert waste into reusable material.
- Pro-environmental political identity: American (Sweetman and Whitmarsh, 2015): An individual's perception that they affiliate with a group of people from a nation (America) whereby that nation prioritises PEB in that country, government, and public affairs.
- Environmental movement identity: Active identity (Dunlap and McCright, 2008): An individual's perception that they belong to a group of people that deliberately engage in social and/or political PEBs.

### What Identity Means for Policy

Based on our research, and interpretations, we propose that policy can utilise the above identities to encourage PEB in three example steps (Hanimann et al., 2015; Udall, 2020):

1. Identify the identity that relates best with the behaviour in question. For example, a cyclist identity did not reduce car use but did increase public transport use for work. Therefore, when interested in encouraging a behaviour, encouraging the appropriate identity is necessary. These levels of nuance are necessary for delivering appropriate messages (Schwartz et al., 2020), but have not always been attained in the existing literature nor practical or policy implications.
2. Only choose pro-PEB identities (Schwartz et al., 2020). For example, some identities (such as a cyclist) relates to PEB positively because the identity is concerned with minimising harm to the planet. However, a motorist identity relates negatively to PEB because it increases harm to the planet and is therefore less appropriate (e.g., Murtagh et al., 2012). As pro-PEB identities are conceptually most alike to PEB, these are most likely to increase PEBs in future.
3. Finally, importantly, decide if the selected identity is an individual and/or the group one and emphasise this clearly. Include messages that are personal and/or people focused to reflect individual and/or group identities, respectively. For example, to encourage a cyclist identity (which would lower CO^2^ compared to driving) you could construct messages based around the following:
  - Individual (me) focused cyclist identity—*Please think about your impact on the planet when going to work—positively imagine yourself cycling*.
  - Group (we) focused cyclist identity—*Please think about our impact on the planet when we go to work—positively imagine yourself with others cycling, and your power as a group to do this (e.g., arrange bike parking spaces at work) as well as the fun while doing this together*

Also, with diminishing time left to make meaningful reductions in greenhouse gas emissions, a broad coalition of distributed approaches is needed alongside behaviour change to mitigate climate change (World Bank, 2015). So far, the consensus in this paper has been on changing individual citizen's behaviour via identity. While we still believe this is imperative, we also acknowledge that systemic change, and policy changing approaches are needed (Buchanan, 1992; Lewis et al., 2020). We can only reach the target of limiting climate change to manageable degrees if all parts of society follow in the same direction; and that includes politicians, administrators, industry, citizens, and education (Szebeko and Tan, 2010; Pieters and Jansen, 2017). Getting all important actors in society to follow this direction is important, and hence research to achieve this is needed (Vandekerckhove et al., 2020).

## Conclusion

Our meta-analysis shows overall identity associates with overall PEB. We found this association by analysing data from the available research on this topic. Before this article, research on the topic has been found to contain a wide range of identity and PEB types without providing a quantitative overview. Our article provided this overview and reveals the areas of identity-PEB research that warrant more attention. Specifically, we show some limitations to theory when understanding the role of identity in PEB research. Additionally, we showed that it is important to distinguish between types of identities and types of PEB to further understand their associations, and then hopefully strengthen them in individuals and groups across the world. Finally, we also show that this field would benefit from much more in-depth and explicit research design considerations, as we found that measurement type (self-reported or observed), setting (lab vs. field), and sample (student vs. non-student) moderated the identity-PEB associations. Thus, both practical interventions, and future research, would benefit from using designs that include representative samples, and suitable measures and settings, to draw robust conclusions. Finally, although our results suggest that the overall Pearson's *r* was 0.34, meaning that identity can explain 11.5% of the variation in PEB (proportion of variance explained can be calculated by squaring the *r* value, i.e., 0.34^2^ = 0.1156 x 100 = 11.56%). While at a first glance this might seem to be a small percentage, considering the complexity of creating a more sustainable world, explaining 11.5% with this identity concept is quite impressive. Also, with millions (or potentially billions) of individuals, who engage (or not) in PEB over many years, even seemingly small changes can accumulate and have a large impact on the environment. Therefore, we maintain that future research would benefit from focusing on people's identities.

## Data Availability Statement

The original contributions presented in the study are included in the article/Supplementary Material, further enquiries can be directed to the corresponding author/s. The dataset and analysis script can be found at: https://osf.io/q7kc4/.

## Author Contributions

AU conceived the project, conducted the method and analysis, and wrote the manuscript. JD, SD, and AS were the supervisors of this project and assisted writing the manuscript. All authors contributed to the article and approved the submitted version.

## Conflict of Interest

The authors declare that the research was conducted in the absence of any commercial or financial relationships that could be construed as a potential conflict of interest.

## Footnotes

^1^https://osf.io/q7kc4/
