# Supplementary material for: How I See Me—A Meta-Analysis Investigating the Association Between Identities and Pro-environmental Behaviour
Source: Front Psychol. 2021 Mar 16;12:582421. doi: 10.3389/fpsyg.2021.582421 (PMC8008126; doi:10.3389/fpsyg.2021.582421)
Supplement: Supplementary file 1 [file Table_1.pdf]

Table S1

Characteristics of Studies Using Identity (ID) Types In Pro-Environmental Behaviour (PEB) - Ordered by Weighted Pearson's r Showing Fisher's Correlation Z, Associated 95% Confidence Intervals (CI) For Studies in a Random-Effects Meta-Analysis Regression

Reference & Study No	Identity Name	PEB related	ID	PEB	Mea-sure	Sample	Size	Setting	Pearson's *r*	Pearson's *r* (weighted)	Sampling Variance	Z-Score	Lower C.I.	Upper C.I.	Recommended to use the identities
**Trump, Connell, & Finkelstein (2015). 3**	Identification with the brand Coca-Cola	Neutral	I	I	S	N	178	F	-.58	-.662	.006	-.797	-.809	-.516	×
**Cook, Kerr, & Moore (2002). 1**	Self-identity with food produced using genetic engineering	-	I	I	S	N	266	F	-.435	-.466	.004	-.505	-.586	-.346	×
**Kaklamanou, Jones, Webb, & Walker (2015). 1**	Pro-environmental (or 'green') identity	+	I	I	S	N	770	F	-.41	-.436	.001	-.467	-.506	-.365	×
**Reese, Berthold, & Steffens (2016). 1**	Superordinate identification with world population, Ingroup identification with developed countries	Neutral	G	G	S	N	90	F	-.115	-.116	.011	-.116	-.322	.091	×
**May, Chang, & Shao (2015). 2**	Moral identification, Organisational identification	Combined	B	G	S	N	149	F	-.115	-.116	.007	-.116	-.276	.045	×
**Steinheider & Hodapp (1999). 1**	Social identity Family, Work	Neutral	G	B	S	N	74	F	-.055	-.055	.014	-.055	-.283	.173	×
**Hernández, Martín, Ruiz, & Hidalgo (2010). 1**	Place identity of island high in environmental protection	+	I	I	S	N	264	F	-.01	-.010	.004	-.01	-.131	.111	×
**Murtagh, Gatersleben, & Uzzell, (2012). 1**	Social identity importance: Member of local community, Parent, Worker; Transport related identity importance: Cyclist, Motorist,	Combined	B	I	S	N	267	F	.004	.004	.004	.004	-.116	.124	?

Reference & Study No	Identity Name	PEB related	ID	PEB	Mea-sure	Sample	Size	Setting	Pearson's *r*	Pearson's *r* (weighted)	Sampling Variance	Z-Score	Lower C.I.	Upper C.I.	Recommended to use the identities
	Pedestrian, Public transport user														
**Graça, Calheiros, & Oliveira (2015). 1**	Dietary identity: Meat eater, Omnivore, Vegan, Vegetarian	Combined	I	I	S	N	1023	F	.01	.010	.001	.010	-.051	.071	?
**Crimston, Bain, Hornsey, & Bastian (2016). 2**	Moral identity: Internalisation, Moral identity: Symbolisation	+	I	B	S	N	119	L	.01	.010	.009	.010	-.170	.190	?
**Fielding, Terry, Masser, & Hogg (2008). 2**	Group identification rural: High, Low	Combined	G	I	S	N	Not applicable	F	.013	.013	Not applicable	.013	Not applicable	Not applicable	?
**Klineberg, McKeever, & Rothenbach (1998). 1**	Self-identified: Ethnicity Anglo, Ethnicity Black, Ethnicity Hispanic, Political ideology Conservative, Political ideology Liberal, Political ideology Moderate	Neutral	I	B	S	N	4005	F	.029	.029	.0002	.029	-.002	.060	?
**Pérez (2009). 1**	Consumers identification with socially responsible insurance company, Corporate social responsibility perceived identity	+	G	I	S	S	296	L	.04	.040	.003	.040	-.074	.154	?
**Reese, Berthold, & Steffens (2016). 2**	Superordinate identification with world population, Ingroup identification with developed countries	Neutral	G	G	O	N	44	F	.05	.050	.024	.050	-.245	.346	?
**Van Dam & Fischer (2015). 2**	Sustainable identity: Connectedness to	+	I	I	O	N	1453	F	.058	.058	.001	.058	.007	.109	√

Reference & Study No	Identity Name	PEB related	ID	PEB	Mea-sure	Sample	Size	Setting	Pearson's *r*	Pearson's *r* (weighted)	Sampling Variance	Z-Score	Lower C.I.	Upper C.I.	Recommended to use the identities
	nature and ethical orientation														
**Lois, Moriano, & Rondinella (2015). 1**	Social identity as cyclist	+	G	I	S	N	595	F	.070	.070	.002	.070	-.010	.151	×
**Fielding, Terry, Masser, & Hogg (2008). 1**	Group identification rural: High, Low	Combined	G	I	S	N	609	F	.087	.087	.002	.087	.007	.166	√
**Zeugner-Roth, Zabkar, & Diamantopoulos (2015). 2**	National identity	Neutral	G	I	S	B	411	F	.100	.100	.002	.100	.003	.197	√
**Rees & Bamberg (2014). 1**	Social identity: Global identification, Sense of neighborhood community	Neutral	G	B	S	N	538	F	.105	.105	.002	.106	.021	.190	√
**Graham-Rowe, Jessop, & Sparks (2015). 1**	Self-identity with fruit and vegetables	+	I	I	S	N	204	F	.117	.117	.005	.118	-.020	.254	×
**Costa-Pinto, Herter, Rossi, & Borges (2014). 1**	**Personal identity, Social identity**	Neutral	B	I	S	N	215	F	.120	.120	.005	.121	-.013	.254	×
**Zeugner-Roth, Zabkar, & Diamantopoulos (2015). 1**	National identity	Neutral	G	I	S	S	411	L	.129	.130	.002	.130	.033	.226	√
**Reese, Berthold, & Steffens (2016). 3**	Superordinate identification with world population, Ingroup identification with developed countries	Neutral	G	G	O	N	69	L	.135	.136	.015	.137	-.100	.372	×
**Crimston, Bain, Hornsey, & Bastian (2016). 3**	Identification with all humanity, Moral identity: Internalisation, Moral identity: Symbolisation	+	B	I	S	N	289	F	.135	.136	.003	.137	.021	.251	√
**Bartels, Reinders, & Van Haaster-De Winter (2015). 1**	Social identification with an	+	G	G	S	S	99	L	.14	.141	.010	.142	-.056	.338	×

Reference & Study No	Identity Name	PEB related	ID	PEB	Mea-sure	Sample	Size	Setting	Pearson's *r*	Pearson's *r* (weighted)	Sampling Variance	Z-Score	Lower C.I.	Upper C.I.	Recommended to use the identities
	environmentally conscious consumer														
**Crimston, Bain, Hornsey, & Bastian (2016). 6**	Moral identity: Internalisation	+	I	G	S	N	99	F	.143	.144	.010	.145	-.053	.341	×
**Barbarossa, Beckmann, De Pelsmacker, Moons, & Gwozdz (2015). 1**	Green self-identity	+	I	I	S	S	2005	L	.15	.151	.0004	.152	.107	.195	√
**Sweetman & Whitmarsh (2015). 1**	Pro-environmental personal identity: British, Pro-environmental political identity: British	+	B	B	S	S	129	L	.16	.161	.008	.163	-.011	.334	×
**Sweetman & Whitmarsh (2015). 3**	Pro-environmental personal identity: British, Pro-environmental political identity: British	+	B	B	S	N	229	F	.19	.192	.004	.195	.063	.322	√
**Loebnitz, Schuitema, & Grunert (2015). 1**	Pro-environmental self-identity	+	I	I	S	N	964	F	.192	.195	.001	.197	.132	.258	√
**Loebnitz & Bröring (2015). 1**	Pro-environmental (or 'green') identity	+	I	I	S	N	498	F	.192	.195	.002	.197	.107	.283	√
**Hwang (2016). 1**	Ethical consumer-identity	+	I	I	S	N	336	F	.204	.207	.003	.210	.100	.314	√
**Schuitema, Anable, Skippon, & Kinnear (2013). 1**	Car-authority identity, Pro-environmental identity	Combined	I	I	S	N	2728	F	.219	.222	.0003	.226	.185	.260	√
**Swim, Zawadzki, Cundiff, & Lord (2014). 1**	Environmental identity: Identification with nature, Place identity with Harris Township an agricultural land	+	I	G	S	S	390	X	.223	.227	.003	.231	.128	.326	√
**Van der Werff, Steg, & Keizer (2014a). 1**	**Environmental self-identity**	+	I	I	S	N	267	F	.23	.234	.004	.239	.114	.354	√

Reference & Study No	Identity Name	PEB related	ID	PEB	Mea-sure	Sample	Size	Setting	Pearson's *r*	Pearson's *r* (weighted)	Sampling Variance	Z-Score	Lower C.I.	Upper C.I.	Recommended to use the identities
**Dunlap & McCright (2008). 1**	Environmental movement identity: Active identity, Neutral identity, Sympathetic identity, Unsympathetic identity	Combined	G	B	S	S	1004	F	.231	.235	.001	.240	.174	.297	√
**Lee (2009). 1**	Self-identity in environmental protection	+	I	I	S	S	2010	X	.24	.245	.0004	.250	.201	.288	√
**Van der Werff, Steg, & Keizer (2014a). 2**	**Environmental self-identity**	+	I	I	S	N	157	F	.24	.245	.006	.250	.088	.401	√
**Shaw, Shiu, & Clarke (2000). 1**	Self-identity with ethical issues	+	I	I	S	N	1471	F	.25	.255	.001	.261	.204	.307	√
**Forsyth, Vugt, Schlein, & Story (2015). 1**	Neighborhood identification, Regional identification	Neutral	G	G	S	N	1131	F	.254	.259	.001	.265	.201	.318	√
**Castro, Garrido, Reis, & Menezes (2009). 1**	Ecological self-identity	+	I	I	S	N	394	F	.26	.266	.003	.273	.167	.365	√
**Matsuba, Pratt, Norris, Mohle, Alisat, & McAdams (2012). 1**	**Environmental identity, Identity maturity**	Combined	I	B	S	N	110	F	.269	.276	.009	.283	.089	.462	√
**Dresner, Handelman, Braun, & Rollwagen-Bollens (2015). 1**	Environmental identity	+	I	I	S	N	165	F	.274	.281	.006	.289	.129	.434	√
**Nigbur, Lyons, & Uzzell (2010). 1**	Neighborhood identification, Self-identity as a recycler	Combined	B	I	B	B	527	L	.279	.287	.002	.295	.202	.372	√
**Bhattacharjee, Berger, & Menon (2014). 2**	**Environmentally conscious identity**	+	I	I	S	N	243	F	.285	.293	.004	.302	.167	.419	√
**Terry, Hogg, & White (1999). 1**	Self-identity: Household recycling, Social identity: Group identification	Combined	B	I	S	S	114	L	.288	.297	.009	.306	.113	.480	√
**Yang, Lu, Zhu, & Su (2015). 3**	Personal identity, Social identity	Neutral	B	I	S	N	165	F	.296	.305	.006	.315	.153	.458	√

Reference & Study No	Identity Name	PEB related	ID	PEB	Mea-sure	Sample	Size	Setting	Pearson's *r*	Pearson's *r* (weighted)	Sampling Variance	Z-Score	Lower C.I.	Upper C.I.	Recommended to use the identities
**Zhang, Van Dijk, Tang, & Berg (2015). 1**	Green space attachment: Place identity	+	I	I	S	B	223	F	.3	.310	.005	.320	.178	.441	√
**Oh & Yoon (2014). 1**	Self-identity	Neutral	I	I	S	N	343	F	.304	.314	.003	.325	.208	.420	√
**Ramkissoon & Mavondo (2015). 1**	Place identity with national park	+	I	I	S	N	339	F	.31	.321	.003	.332	.214	.427	√
**Nigbur, Lyons, & Uzzell (2010). 2**	Neighborhood identification, Self-identity as a recycler	Combined	B	I	S	N	264	F	.315	.326	.004	.338	.205	.447	√
**Tonge, Ryan, Moore, & Beckley, (2015). 1**	Place identity with Ningaloo Marine Park	+	I	I	S	N	372	F	.317	.328	.003	.341	.226	.430	√
**Kiesling & Manning (2010). 1**	Environmental identity, Environmental gardening identity: Connection to wild, Natural function, Pesticide avoidance, Willingness to engage with natural processes, Worldview	+	I	I	S	S	466	F	.320	.331	.002	.344	.240	.422	√
**Van der Werff, Steg, & Keizer (2013). 2**	Environmental self-identity	+	I	I	S	N	45	F	.33	.343	.024	.357	.051	.635	√
**White, Smith, Terry, Greenslade, & McKimmie (2009). 2**	Social identity: Group identification	Neutral	G	I	S	S	175	L	.335	.348	.006	.364	.200	.497	√
**Costa-Pinto, Nique, Herter, & Borges (2016). 1**	**Personal identity, Social identity**	Neutral	B	I	S	S	153	F	.340	.349	.007	.364	.190	.507	√
**Bissonnette & Contento (2001). 1**	Perceived self-identity as health-conscious & environmentally concerned	+	I	I	S	S	70		.340	.354	.015	.370	.119	.588	√
**Van der Werff, Steg, & Keizer (2013). 3**	**Environmental self-identity**	+	I	I	S	B	70	F	.34	.354	.015	.370	.120	.588	√

Reference & Study No	Identity Name	PEB related	ID	PEB	Mea-sure	Sample	Size	Setting	Pearson's *r*	Pearson's *r* (weighted)	Sampling Variance	Z-Score	Lower C.I.	Upper C.I.	Recommended to use the identities
**Leung, Koh, & Tam (2015). 1**	Global identity, Local identity	Neutral	G	G	S	N	659	F	.343	.358	.002	.374	.282	.434	
**Halpenny (2010). 1**	Place Identity-Affect to Point Pelee National Park	+	I	I	S	S	354	L	.355	.371	.003	.390	.267	.475	√
**Tam (2013). 1**	Environmental identity	+	I	B	S	S	322	L	.36	.377	.003	.396	.268	.486	√
**Yazdanpanah & Forouzani (2015). 1**	Self-identity with organic food	+	I	I	S	B	389	F	.36	.377	.003	.396	.278	.476	√
**Alisat & Riemer (2015). 1**	Environmental identity	+	I	G	S	S	281	F	.36	.377	.004	.396	.260	.494	√
**Trump, Connell, & Finkelstein (2015). 2**	Identification with a beloved candy brand: Reese's	Neutral	G	I	S	N	98	F	.37	.388	.011	.410	.190	.586	√
**Nilsson, Andersson, & Bergstad (2015). 1**	Group identity with department	Neutral	G	I	S	N	71	F	.374	.393	.015	.416	.161	.626	√
**Sweetman & Whitmarsh (2015). 2**	Pro-environmental personal identity: American, Pro-environmental political identity: American	+	B	B	S	N	333	F	.393	.415	.003	.441	.307	.522	√
**Memery, Angell, Megicks, & Lindgreen (2015). 1**	Local identity	Neutral	G	I	S	N	1223	F	.393	.416	.001	.442	.360	.472	√
**Bartels & Onwezen (2014). 1**	Social identification with organic consumer	+	G	I	S	S	1006	F	.4	.424	.001	.452	.362	.485	√
**Stets & Biga (2003). 1**	Role identity Gender, Self-identity with organic food: Environment identity, Commitment, Prominence, Salience	Combined	I	B	S	N	365	F	.4	.424	.003	.452	.321	.526	√
**Sparks & Shepherd (1992). 1**	Self-identity as a health-conscious consumer, Self-identity	+	I	I	S	B	261	F	.403	.427	.004	.456	.305	.548	√

Reference & Study No	Identity Name	PEB related	ID	PEB	Mea-sure	Sample	Size	Setting	Pearson's *r*	Pearson's *r* (weighted)	Sampling Variance	Z-Score	Lower C.I.	Upper C.I.	Recommended to use the identities
	with green consumerism														
**Mannetti, Pierro, & Livi (2004). 1**	Identity similarity with typical recyclers	+	G	I	S	N	230	F	.41	.436	.004	.467	.306	.565	√
**Van der Werff, Steg, & Keizer (2014b). 1**	Environmental self-identity		I	I	S	S	335	F	.41	.436	.003	.467	.329	.543	√
**Watson, Johnson, Hegtvedt, & Parris (2015). 1**	Environmental identity	+	I	B	S	S	243	X	.412	.438	.004	.470	.312	.564	√
**Reese & Kohlmann (2015). 1**	Social identity: Global identification	Neutral	G	I	B	N	68	F	.415	.442	.015	.474	.204	.679	√
**Juneman & Rufaedah (2013). 1**	National identity: Indonesian	Neutral	G	B	S	N	262	F	.427	.456	.004	.492	.335	.577	√
**Forsyth, Vugt, Schlein, & Story (2015). 2**	Neighborhood identification, Regional identification	Neutral	G	G	S	N	57	F	.447	.481	.019	.525	.222	.741	√
**Lee, Levy, & Yap (2015). 1**	Place identity: Attachment, cohesion, commitment, continuity with personal past, perception of familiarity	Neutral	I	B	S	B	561	F	.46	.497	.002	.546	.415	.580	√
**Barth, Jugert, & Fritsche (2016). 2**	Collective efficacy, Descriptive norms, Injunctive norms, Provincial norms, Subjective norms	Neutral	G	I	S	N	548	F	.462	.500	.002	.549	.416	.584	√
**Costa-Pinto, Nique, Herter, & Borges (2016). 2**	Personal identity, Social identity	Neutral	B	I	S	N	119	F	.470	.509	.009	.561	.329	.688	√
**Van der Werff, Steg, & Keizer (2013). 1**	Environmental self-identity	+	I	I	S	S	138	L	.47	.510	.007	.563	.343	.677	√

Reference & Study No	Identity Name	PEB related	ID	PEB	Mea-sure	Sample	Size	Setting	Pearson's *r*	Pearson's *r* (weighted)	Sampling Variance	Z-Score	Lower C.I.	Upper C.I.	Recommended to use the identities
**May, Chang, & Shao (2015). 1**	Moral identification, Moral identity	+	I	I	S	N	216	F	.475	.517	.005	.572	.383	.650	√
**Bobek, Hageman, & Radtke (2015). 1**	Organisational fit with the public accounting firm, Public interest orientation, Role in shaping and maintaining the ethical environment	Combined	B	B	S	N	139	F	.497	.545	.007	.612	.379	.712	√
**McCright & Dunlap (2015). 1**	Environmental movement identity: Active participant, Neutral, Sympathetic & not active participant, Unsympathetic; Self-identified environmentalist: Not one, Not strong, Strong	Combined	B	I	S	N	1004	F	.5	.549	.001	.617	.487	.611	√
**Hall-Phillips, Park, Chung, Anaza, & Rathod (2016). 1**	Consumer-Social venture identification, Identity attractiveness, Identity distinctiveness, Identity similarity	Combined	B	G	S	N	633	F	.5	.549	.002	.617	.471	.627	√
**Van Dam & Fischer (2015). 1**	Sustainable identity: Connectedness to nature and ethical orientation	+	I	I	S	N	229	F	.509	.561	.004	.635	.432	.691	√
**Brügger, Kaiser, & Roczen (2011). 1**	Environmental identity	+	I	I	S	S	1309	L	.54	.604	.001	.700	.550	.658	√
**Van der Werff, Steg, & Keizer (2014b). 3**	**Environmental self-identity**	+	I	I	S	N	150	L	.548	.615	.007	.717	.455	.775	√
**Lu, Rahman, & Chi (2016). 1**	Genetically modified wine identity (negative stimulus)	Neutral	I	I	S	N	199	F	.562	.636	.005	.752	.497	.775	√

Reference & Study No	Identity Name	PEB related	ID	PEB	Mea-sure	Sample	Size	Setting	Pearson's *r*	Pearson's *r* (weighted)	Sampling Variance	Z-Score	Lower C.I.	Upper C.I.	Recommended to use the identities
**Bartels & Hoogendam (2011). 1**	Social identification with environmentally conscious consumer, Social identification with organic consumer	+	G	I	S	N	961	L	.57	.648	.001	.771	.584	.711	√
**Tam (2013). 2**	Allo-inclusive identity: Inclusion of people, animals, & inanimate entities, Environmental identity	+	I	B	S	S	185	L	.57	.648	.005	.771	.503	.792	√
**Davis, Le, & Coy (2011). 1**	Environmental identity	+	I	I	S	S	248	F	.585	.670	.004	.811	.546	.794	√
**Dono, Webb, & Richardson (2010). 1**	Social identity: Dislike of group & no identification with (social) group of environmentalists, Group identification with (social) group of environmentalists, Group identification with environmentalist	Combined	G	B	S	N	131	F	.585	.670	.008	.811	.499	.841	√
**Khare (2015b). 1**	Green self-identity in environmental protection	+	I	I	S	N	490	F	.595	.685	.002	.839	.597	.774	√
**Rex, Lobo, & Leckie (2015). 1**	Internal ethics	+	I	I	S	N	511	F	.61	.709	.002	.885	.622	.796	√
**Khare (2015a). 1**	Green self-identity in environmental protection	+	I	I	S	N	490	F	.628	.739	.002	.948	.650	.827	√
**White & Hyde (2012). 1**	Self-identity as a recycler	+	I	I	S	N	148	F	.63	.741	.007	.954	.580	.903	√
**Chatzidakis, Kastanakis, & Stathopoulou (2016). 1**	Internal ethics	+	I	I	S	S	517	L	.663	.798	.002	1.093	.712	.884	√

Reference & Study No	Identity Name	PEB related	ID	PEB	Mea-sure	Sample	Size	Setting	Pearson's *r*	Pearson's *r* (weighted)	Sampling Variance	Z-Score	Lower C.I.	Upper C.I.	Recommended to use the identities
**Andorfer & Liebe (2013). 1**	Fair Trade consumer identity	+	I	I	S	S	556	F	.669	.808	.002	1.123	.725	.892	√
**Hinds & Sparks (2008). 1**	Environmental identity	+	I	I	S	N	199	F	.67	.811	.005	1.129	.672	.950	√
**Dermody, Hanmer-Lloyd, Koenig-Lewis, & Zhao (2015). 1**	Pro-environmental self-identity	+	I	I	S	N	2062	F	.67	.811	.0004	1.129	.768	.854	√
**Whitmarsh & O'Neill (2010). 1**	Behaviour generic self-identity: Pro-environmental, Behaviour specific self-identity: Carbon offsetting	+	I	B	S	S	551	F	.678	.826	.002	1.175	.742	.909	√
**Bamberg, Rees, & Seebauer (2015). 1**	Group identity with Transition Towns	+	G	G	S	S	652	F	.74	.950	.002	1.837	.874	1.027	√
**Fielding, McDonald, & Louis (2008). 1**	Self-identity with environmental activism, Social identity: Group membership of an environmental group	+	B	G	S	N	169	F	.77	1.020	.006	Not applicable	.870	1.171	√
**Lokhorst, Hoon, Le Rutte, & de Snoo (2014). 1**	Self-identity in private nature conservation	+	I	I	S	N	94	F	.79	1.071	.011	Not applicable	.869	1.274	√
**Overall Pearson's *r***	**All The Above Identities Combined**	**Combined**	**B**	**B**	**B**	**B**	**49860**	**B**	**.31**	**.34**	**.01**	**12.16**	**.29**	**.40**	Not applicable

***Note*. Pro-environmental behaviour (PEB) related column = positively thematically related to the PEB definition (+), negatively thematically related to the PEB definition (-), neither positively nor negatively, namely, neutrally thematically related to the PEB definition (Neutral), thematically related to the PEB definition in more than one way, +, and/or – and/or neutral (combined). Identity (ID) column: individual identity (I), group identity (G) or both (B) focused. Pro-environmental behaviour (PEB) column: Individual PEB (I), group PEB (B) or both (B) focused. Measure column: PEB self-report (S), PEB observed (O), or both (B). Setting column: Lab (L), field (F) or unknown (X). Sample column: Student (S), non-student (N), or both (B). C.I. = Confidence intervals. Pearson's *r* column = Estimate as a regression coefficient; Pearson's *r* (weighted) column = Estimate as a regression coefficient weighted by its sample size. Final column (16): Yes (√), no (×), perhaps (?).**
